# Discovery of Lead‐Free Perovskites for High‐Performance Solar Cells via Machine Learning: Ultrabroadband Absorption, Low Radiative Combination, and Enhanced Thermal Conductivities

**DOI:** 10.1002/advs.202103648

**Published:** 2021-12-14

**Authors:** Xia Cai, Yiming Zhang, Zejiao Shi, Ying Chen, Yujie Xia, Anran Yu, Yuanfeng Xu, Fengxian Xie, Hezhu Shao, Heyuan Zhu, Desheng Fu, Yiqiang Zhan, Hao Zhang

**Affiliations:** ^1^ School of Information Science and Technology Fudan University Shanghai 200433 China; ^2^ Center of Micro‐Nano System School of Information Science and Technology Fudan University Shanghai 200433 China; ^3^ Key Laboratory of Micro and Nano Photonic Structures (MOE) and Department of Optical Science and Engineering Fudan University Shanghai 200433 China; ^4^ School of Science Shandong Jianzhu University Jinan Shandong 250101 China; ^5^ College of Electrical and Electronic Engineering Wenzhou University Wenzhou 325035 China; ^6^ Department of Electronics & Materials Sciences Faculty of Engineering, & Department of Optoelectronics and Nanostructure Science Graduate School of Science and Technology Shizuoka University Hamamatsu 432‐8561 Japan; ^7^ Yiwu Research Institute of Fudan University Chengbei Road Yiwu City Zhejiang 322000 China

**Keywords:** density‐functional theory, hybrid organic–inorganic perovskites, lead‐free double perovskites, machine learning, photovoltaics

## Abstract

Exploring lead‐free candidates and improving efficiency and stability remain the obstacle of hybrid organic‐inorganic perovskite‐based devices commercialization. Traditional trial‐and‐error methods seriously restrict the discovery especially for large search space, complex crystal structure and multi‐objective properties. Here, the authors propose a multi‐step and multi‐stage screening scheme to accelerate the discovery of hybrid organic‐inorganic perovskites A_2_BB′X_6_ from a large number of candidates through combining machine learning with high‐throughput calculations for pursuing excellent efficiency and thermal stability in solar cells. Followed by a series of screenings, the structure‐property relationships mapping A_2_BB′X_6_ properties are built and the predictions are close to reported experimental results. Successfully, four experimental‐feasibly candidates with good stability, high Debye temperature and suitable band gap are screened out and further verified by density‐functional theory calculations, in which the predicted efficiency for three lead‐free candidates ((CH_3_NH_3_)_2_AgGaBr_6_, (CH_3_NH_3_)_2_AgInBr_6_ and (C_2_NH_6_)_2_AgInBr_6_) achieves 20.6%, 19.9% and 27.6% due to ultrabroadband absorption region ranging from UVC to IRC with excitonic radiative combination rates as low as 10 ps, large or intermediate polarons form with properties similar to CH_3_NH_3_PbI_3_ and the calculated thermal conductivities are 5.04, 4.39 and 5.16 Wm^−1^K^−1^, respectively, with Debye temperatures larger than 500 K, beneficial for suppression of both nonradiative combination and heat‐induced degradation.

## Graphical abstract

A multi‐step screening scheme is proposed to accelerate the discovery of potential perovskite A_2_BB′X_6_ through combining machine learning with high‐throughput calculations for the target of excellent efficiency and thermal stability in solar cells. Among final candidates, the predicted efficiency for three lead‐free candidates achieves 20.6%, 19.9% and 27.6%, and the calculated thermal conductivities are 5.04, 4.39 and 5.16 Wm^−1^K^−1^, respectively.

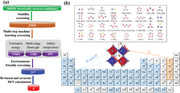

## 1 Introduction

Hybrid organic–inorganic perovskites (HOIPs) represented by CH_3_NH_3_PbX_3_ as promising next‐generation photovoltaic materials have attracted great attention in recent years and have revolutionized solar cells since in 2009 Kojima et al. first successfully reported with a power conversion efficiency (PCE) of 3.8%.^[^ ^1^ ^]^ The PCE of HOIPs‐based photovoltaic system has increased to 25.5% in only 10 years.^[^ ^2^ ^]^ In addition to further improving PCE of HOIPs, many efforts have been done, such as 2D/3D mixed dimensional perovskite,^[^ ^3^, ^4^, ^5^ ^]^ charge transport layer modification,^[^ ^6^, ^7^, ^8^ ^]^ interlayer insertion,^[^ ^9^, ^10^ ^]^ and post encapsulation^[^ ^11^ ^]^ to restrain HOIPs decomposing into Pb^2 +^ composites. However, due to the limited impacts of these works on material intrinsic stability, Pb^2 +^ eventually poisons the earth after a long period time scale.^[^ ^12^, ^13^, ^14^ ^]^ Pb substitution is a widely accepted concept to wipe out the toxic issue from HOIPs. Another concern is the intrinsic instability of perovskite. Industry market standard requires solar cells to work steady over 25 years in ambient.^[^ ^15^ ^]^ Light‐ and heat‐induced degradation have been integrally studied and proved to be two major issues in HOIPs.^[^ ^16^, ^17^, ^18^ ^]^ For example, CH_3_NH_3_PbI_3_ can be easily degraded due to the light‐induced reaction, that is, CH_3_NH_3_PbI${}_{3}\overset{hv}{⇄}$ PbI_2_ + CH_3_NH_2_ ↑ + HI↑,^[^ ^19^ ^]^ and heat‐induced degradation due to the thermal conductivity as low as 0.5 Wm^−1^ *K* ^−1^ at room temperature,^[^ ^20^ ^]^ respectively. After baking at over 150 ^*o*^C, HOIPs easily go through an endothermic reaction and decompose into its components like PbI_2_ and HI.^[^ ^21^, ^22^ ^]^ The general solutions to thermal issue in device are to dissipate accumulated heat efficiently by adding heat‐dissipation layers, while the complexity of device fabrication process increases.^[^ ^23^, ^24^ ^]^ Generally, in materials, Debye temperature $\theta_{D}\propto n{\left(\frac{\rho }{M}\right)}^{1/3}v_{m}$, in which *n*, ρ, *M*, and *v*_*m*_ are the number of atoms per formula unit, the crystal structure's density, molar mass, and average sound velocity, respectively, plays a key role in proxy for structure rigidity and directly relates to the thermal conductivity. If perovskite material possesses a high Debye temperature, generally leading to high thermal conductivity, which is beneficial to rapidly dissipate heat and conducive to the thermal stability of devices.^[^ ^25^, ^26^ ^]^ High Debye temperature may decrease the non‐radiative recombination in solar cells as well, due to the high phonon energies. Therefore, HOIPs with the merits of lead free and high Debye temperature should be explored to pursuit the goal of excellent efficiency and thermal stability in perovskite solar cells. The double‐perovskite A_2_BB′X_6_ named HOIDP with A the molecular cation, B/B′ the metal cation, and X the anionic bridging ligand, has emerged as a new class promising lead‐free HOIPs, due to diverse electronic structures and multiple material selections. However, currently, most of the works on lead‐free A_2_BB′X_6_ focus on inorganic materials, for example, Cs_2_AgBiBr_6_, which mainly suffers from the following problems: 1) its absorption is usually below 650 nm^[^ ^27^ ^]^ which prevents the charge generation; 2) low thermal conductivity of A_2_BB′X_6_ leads to high working temperature of devices; 3) complex structures make it difficult to maintain the lattice stability; 4) the formation of small polarons or self‐trapped excitons limits mobilities, thus suppressing diffusion/drift lengths; 5) multiple material options lead trial‐and‐error method work labor‐intensive and time‐consuming. Therefore, the search for new A_2_BB′X_6_ HOIDPs with excellent performance and good stability has become an urgent task.

Compared with the traditional trial‐and‐error approach to discover new materials based on scientists' physical and chemical intuition, the emergence of advanced techniques, such as high‐throughput computation based on density‐functional theory (DFT) method, has greatly accelerated the search process.^[^ ^28^, ^29^, ^30^, ^31^, ^32^ ^]^ However, the inherent complexity of materials severely hinders its efficiency due to the large‐scale real‐world chemical space. Fortunately, the rapid advances in Materials Genome Project^[^ ^33^ ^]^ and artificial intelligence technologies offer exciting hope for this dilemma.^[^ ^34^, ^35^, ^36^, ^37^, ^38^, ^39^, ^40^, ^41^, ^42^, ^43^, ^44^, ^45^, ^46^, ^47^, ^48^, ^49^ ^]^ Unlike first‐principles approaches, machine learning (ML) technique can be used smoothly to rapidly predict one or more target properties without relying on numerically solving complex systems of quantum mechanical equations, once suitable material database is established and efficient model is selected, which requires several orders of magnitude fewer computational resources than traditional method. Recently, ML technique has made significant progress in the design of rational materials, such as photovoltaic materials,^[^ ^34^, ^35^, ^36^, ^37^, ^38^, ^39^ ^]^ catalysts,^[^ ^40^, ^41^, ^42^ ^]^ lithium batteries,^[^ ^43^ ^]^ and so on. Notably, many materials predicted by ML technique have been synthesized experimentally and have shown excellent properties. These successful attempts^[^ ^44^, ^45^, ^46^, ^47^, ^48^, ^49^ ^]^ have demonstrated that intelligent ML technique bypassing intense DFT calculations or experimental trials can enable low‐cost, fast, and highly accurate prediction of target material properties, greatly accelerating material discovery.

In this work, we develop a multi‐step material screening scheme to accelerate the discovery of new lead‐free HOIDPs with enhanced thermal stability for high‐performance solar cells, by combining high‐throughput DFT calculations with ML technique. The perovskite stability, band gap, and Debye temperature are selected as three target properties to step‐wise screen the chemical space. As the fundamental step for global search of possible HOIDPs candidates, a database containing 180 038 electrically neutral compounds is firstly screened from a full chemical space of elemental combinations in the periodic table based on 32 organic cations, and then the condition of structure stability is used to screen out candidates with unstable structure. Third, different ML models are built for multi‐goal and multi‐stage screen with high accuracy and the importance of relevant features on learning goal is analyzed. Based on ML‐predicted results some orthorhombic‐like HOIDPs candidates for light‐harvesting applications stand out, and Br‐based and environment‐friendly candidates are selected for further DFT validation. Finally, four lead‐free HOIDPs with enhanced thermal stability are picked out as promising solar cell materials with appropriate band gap and high Debye temperature.

## 2 Results

### 2.1 Material Screening Framework

**Figure 1:**
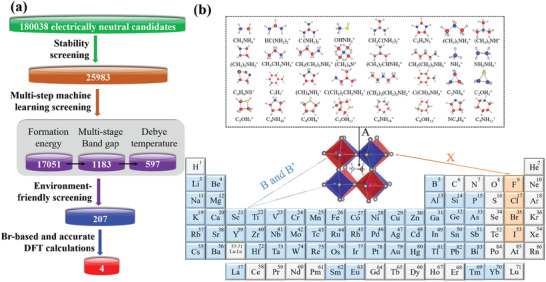
The left chart for the process of discovering novel HOIDPs according to the combination of ML and DFT calculation for photovoltaic application and the right chart for the composition and structure of perovskites in prediction set. Here, the combinations of 32 monovalent organic molecular cations for A site, 9 monovalent, 49 divalent, and 35 trivalent B/B′‐site cations, and 4 X‐site anions cross the periodic table produce the set of unexplored HOIDPs candidates. In the condition of charge neutrality, 180038 initial candidates are obtained. Then through the stability condition and ML method, 597 HOIDPs suitable for solar cells are chosen. Finally, electronic and other properties of these chosen candidates are further verified by DFT calculations, and four ideal HOIDPs with good quality are finally selected.

In this work, we propose to use the multi‐step material screening approach to discover novel hybrid organic–inorganic double perovskites for photovoltaic application with good stability, high Debye temperature, and suitable band gap. The workflow of screening processes is schematically illustrated in **Figure** **1**a. In the first screening process of our material‐search scheme, the HOIDP compounds must satisfy the charge neutrality criterion, and then tolerance factor and octahedral factor are used to evaluate the structural stability of perovskite candidates. A large chemical space for HOIDP candidates is generated subsequently.

The procedure of machine‐learning screening for the generated large chemical space includes three parts: first, a regression model is built to obtain the average formation energy per atom of HOIDPs, in order to judge the chemical stability of each compound. Second, three regression models are built to predict the band gap of HOIDPs to ensure the accuracy of the screening, which can help identify candidates with the appropriate band gap. In the parallel step, another regression model is built to pick the candidates with high Debye temperature to select candidates with large thermal conductivity and good thermal stability. A much smaller set of HOIDP candidates is generated after the machine‐learning screening based on the formation‐energy, band‐gap and Debye‐temperature models. Finally, the accuracy of the predicted small set is further verified by DFT calculation, including computing the electronic property, formation energy, Debye temperature and other properties for screening candidates.

### 2.2 Dataset and Modeling: High‐Throughout Calculation of Debye Temperature

Generally, in order to train a good ML model, a high‐quality training dataset is required. Data diversity is important for the prediction accuracy of ML models. In addition to inert gas and radioactive elements, the elements utilized in our training and test sets cover almost the entire periodic table, which should be reliable enough to realize accurate ML model. The input dataset of this work composed of 4456 HOIDPs is obtained from high‐throughput DFT calculations.^[^ ^31^, ^32^ ^]^ All the selected compounds possess the double perovskite structures with the chemical formula of A_2_BB′X_6_. As shown in Figure 1b, the halogen atoms X (X=F, Cl, Br, I) occupy the vertices of the corner‐sharing BX_6_ or B′X_6_ octahedron. The elements of B and B′ can be same or different, and the number of different B‐site and B′‐site cations in our dataset is 20. 16 kinds of monovalent A^+^‐site cations are filled in the cavity formed by the adjacent octahedrons. In order to define the feature vector of each A_2_BB′X_6_ compound, 95 features based on the main characteristics of elements listed in Table S1, Supporting Information, are utilized to represent, which can be obtained directly from the periodic table to facilitate ML prediction.

The calculated formation energies of 4456 HOIDPs used as the dataset for building formation‐energy ML model are shown in Figure S1a, Supporting Information, which plots the relation between Goldschmidt tolerance factor (*T*_*f*_) and formation energy. The *T*_*f*_ is defined by $\frac{R_{A}+R_{X}}{\sqrt{2}\left(R_{\bar{B}}+R_{X}\right)}$, with *R*_*A*_ and *R*_*X*_ representing the ionic radius of A cation and X anion, respectively, and $R_{\bar{B}}$ representing the average ionic radius of the B and B′ cations. Effective radii^[^ ^50^ ^]^ and Shannon's ionic radii^[^ ^51^ ^]^ are adopted for molecular ions and atomic ions, respectively. As shown in Figure S1a, Supporting Information, most of the chemically stable HOIDPs in the dataset with the formation energies less than 0 eV atom^‐1^ possess the *T*_*f*_ values ranging from 0.80 to 1.25, which is consistent with the chemical tuition for perovskites that the appropriate tolerance factor *T*_*f*_ should be in the range between 0.8 and 1.2.^[^ ^52^, ^53^, ^54^, ^55^ ^]^

The dependence of *T*_*f*_ on Perdew–Burke–Ernzerhof (PBE) band gap of 4456 HOIDPs are shown in Figure S1b, Supporting Information, in which all the compounds can be divided into three categories: metals (zero band gap, 3533), semiconductors (band gap between 0 and 3.5 eV, 776), and insulators (band gap lager than 3.5 eV, 147). We extract 425 compounds with direct band gap and verify the band gap by PBE‐DFT calculation, to constitute the dataset for further modeling. Considering that PBE method usually underestimates the band gap of semiconductor, we also build the band‐gap ML model based on the reported 663 compounds with accurate Heyd–Scuseria–Erzenhof (HSE) band gap. Involving all the 95 features for the PBE/HSE band‐gap training dataset does not mean characterizing perovskites well, since too many features sometimes increase the complexity of ML model (called “curse of dimension”), leading to over fitting. Thus, we also implement the process of feature selection to simplify the inputs of model.

As we know, Debye temperature (Θ_*D*_) is the most reliable proxy for structural rigidity, and the materials with higher Θ_*D*_ tend to have higher‐energy phonon modes that decrease the probability of non‐radiative relaxation,^[^ ^25^, ^26^ ^]^ which is beneficial for the enhancement of quantum coherence and device performances in HOIDP‐based solar cells. Generally, above Θ_*D*_, all phonons in materials are excited. According to the slack model,^[^ ^56^ ^]^ the lattice thermal conductivity in materials can be estimated by $\kappa_{L}=A\times \frac{\bar{M}\Theta_{D}^{3}\delta }{\gamma^{2}n^{2/3}T}$, where $\bar{M}$ is the average mass per atom in the crystal, δ^3^ is the volume per atom, γ is the Grüneisen parameter, *n* is the number of atoms in the primitive unit cell, and *A* is a constant determined by γ, that is, $A=\frac{2.43\cdot 10^{-8}}{1-0.514/\gamma +0.228/\gamma^{2}}$. The Grüneisen parameter (γ) can been calculated by $\gamma =\frac{3}{2}\left(\frac{1+v_{p}}{2-3v_{p}}\right)$,^[^ ^57^ ^]^ where $v_{p}=\frac{1-2{\left(v_{T}/v_{L}\right)}^{2}}{2-2{\left(v_{T}/v_{L}\right)}^{2}}$ according to longitudinal (*v*_*L*_) and transverse (*v*_*T*_) sound velocity found in Equation (17). Therefore, large Debye temperature leads to high κ_*L*_. Due to computational intensity, we only calculate Debye temperature for the 425 HOIDP compounds with direct band gap.

The ML models for formation energies per atom, band gap and Debye temperature built by seven ML algorithms are shown in Figure S2, Supporting Information, in which black dots are the values of determination coefficient (R^2^), red dots represent mean square error (MSE), and blue dots are mean absolute error (MAE). The higher R^2^, the smaller MSE, the smaller MAE, and the better performance of the trained model. As shown in Figure S2a– d, Supporting Information, among the seven regression algorithms, the GBR algorithm performs best among these models, which gives R^2^/MSE/MAE of 0.990/0.021/0.013 eV atom^‐1^, 0.920/0.307/0.241 eV, 0.870/0.384/0.278 eV, and 0.990/16.741/11.737 K for models of formation energy, PBE band gap, HSE band gap, and Debye temperature, respectively.

### 2.3 Basic Screening

Herein, the common valence of element is considered to use to calculate the charge neutrality of each candidate. In this way, we first screen out a large number of non‐neutral compounds from the possible candidate compounds. Monovalent A^+^‐site organic cation, divalent B^2 +^‐site and B′^2 +^‐site cations and monovalent X^−^‐site halogen anion or monovalent A^+^‐site organic cation, monovalent B^+^‐site cation, trivalent B′^3 +^‐site cation, and monovalent X^−^‐site halogen anion are collected to generate possible HOIDP candidates. Here, 32 different kinds of monovalent organic molecular cations including CH_3_NH${}_{3}^{+}$, HC(NH${}_{{2)}_{2}}^{+}$, C(NH${}_{{2)}_{3}}^{+}$, OHNH${}_{3}^{+}$, CH_3_C(NH${}_{{2)}_{2}}^{+}$, C_3_H_5_N${}_{2}^{+}$, (CH_3_)_2_NH${}_{2}^{+}$, (CH_3_)_3_NH^+^, (CH_2_)_3_NH${}_{2}^{+}$, CH_3_CH_2_NH${}_{3}^{+}$, CH_3_CH_2_CH_2_NH${}_{3}^{+}$, (CH_3_)_4_N^+^, (CH_3_)_2_CHNH${}_{3}^{+}$, CH_3_(CH_2_)_3_NH${}_{3}^{+}$, NH${}_{4}^{+}$, NH_2_NH${}_{3}^{+}$, C_3_H_4_NS^+^, C_7_H${}_{7}^{+}$, (CH)_4_NH${}_{2}^{+}$, C(CH_3_)_2_CH_2_NH${}_{2}^{+}$, (CH_3_)_2_(CH_2_)_2_NH${}_{2}^{+}$, C(CH)_5_NH${}_{3}^{+}$, C_2_NH${}_{6}^{+}$, C_2_OH${}_{5}^{+}$, C_3_OH${}_{7}^{+}$, C_4_NH${}_{10}^{+}$, C_4_OH${}_{9}^{+}$, C_5_OH${}_{12}^{+}$, C_6_NH${}_{14}^{+}$, C_6_OH${}_{13}^{+}$, NC_4_H${}_{8}^{+}$, C_5_NH${}_{12}^{+}$, are used in A^+^ site, which are shown in the top of Figure 1b. Meanwhile, 9 monovalent B^+^‐site cations (Li, Na, K, Rb, Cs, Cu, Ag, Au, Tl), 49 divalent B^2 +^‐site and B′^2 +^‐site cations (Be, Mg, Sr, Ba, La, Ti, Zr, Hf, V, Nb, Ta, Cr, Mo, W, Mn, Tc, Re, Fe, Ru, Os, Co, Rh, Ir, Ni, Pd, Pt, Cu, Ag, Zn, Cd, Hg, Si, Ge, Sn, Pb, Ca, Eu, Tm, Yb, Sm, P, Sc, As, Y, In, Sb, Au, Tl, Bi) and 35 trivalent B′^3 +^ site cations (Sc, Y, La, Ti, Zr, Hf, V, Nb, Ta, Cr, Mo, W, Mn, Tc, Re, Fe, Ru, Os, Co, Rh, Ir, Ni, Pd, Pt, Cu, Ag, Au, B, Al, Ga, In, Tl, As, Sb, Bi) are employed. The list of atoms at B‐, B′‐, and X‐site are shown in the periodic table of Figure 1b. According to the rule of charge neutrality, 180 038 electrically neutral HOIDPs are firstly generated.

Then, we conduct further screening regarding the structural formability of the firstly generated candidates, based on *T*_*f*_ and the octahedral factor (*O*_*f*_)^[^ ^58^ ^]^ given by $\frac{R_{\bar{B}}}{R_{X}}$. As mentioned above, to form a stable perovskite structure, generally, *T*_*f*_ and *O*_*f*_ have values of 0.8 ≈ 1.2 and 0.4 ≈ 0.7, respectively.^[^ ^52^, ^53^, ^54^, ^55^ ^]^ Thus, 25983 compounds with structural formability are selected from the 180 038 charge neutral candidates, which can be used to conduct further prediction with ML method.

### 2.4 Model Inference

#### 2.4.1 Chemical Stability: Formation Energy

**Figure 2:**
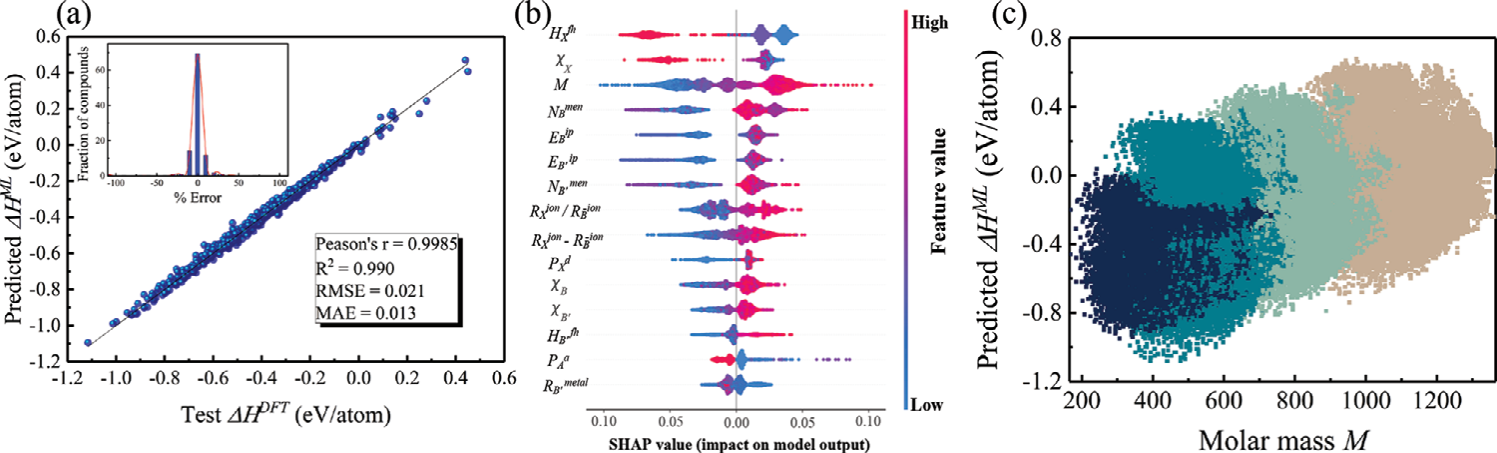
The chart results from formation‐energy ML model. a) Actual formation energy per atom △*H* ^*DFT*^ in the test set and predicted formation energy per atom △*H* ^*ML*^. Pearson coefficient (r), Coefficient of determination (R^2^), mean squared error (MSE), and mean absolute error (MAE) are computed to estimate the prediction errors. The ideal line shown as red line is about ideal prediction result and the fit line shown as dark line is about actual prediction result. These two lines basically coincide. The inset is the fraction of compounds according to their percent error between predicted △*H* ^*ML*^ and △*H* ^*DFT*^. The red curve shows the trend. b) The feature importance ranking produced from the gradient boosting regression and SHAP library, showing the elemental properties in descending order of importance. All samples in the dataset are presented and a point in the graph is corresponding to a sample. The *x*‐axis labeled as the SHAP value represents the impact of features on formation energy. The red and blue color indicate high and low values of a given feature, respectively. c) The prediction of formation energy for all electrically neutral candidates with molar mass of compound.

The comparison of predicted formation energies with those in test dataset for GBR model is shown in **Figure** **2**a, which are basically coincident. The inset shows the percent error between predicted △*H* ^*ML*^ and △*H* ^*DFT*^ of 95% compounds is less than 10%. The SHAP (Shapley Additive exPlanations) method is used to further interpret our model,^[^ ^59^ ^]^ which is a generalized metric for feature importance and employ the game‐theory‐based Shapley values to obtain the contribution of each feature to the model's output. The top 15 features are displayed in Figure 2b, which are listed by the ranking importance, that is, the heat of formation of X site ($H_{X}^{fh}$), the electronegativity of X site (χ_*x*_), molar mass (*M*), the Mendeleev's number of B site ($N_{B}^{men}$), and the first ionization energy of B and B′ sites ($E_{B}^{ip}$ and $E_{B^{\prime }}^{ip}$).

Overall, as shown in Figure 2b, instead of the traditional chemical tuition of *T*_*f*_ and *O*_*f*_ for structural formability of perovskites, the features from B, B′ and X sites, that is, $H_{X}^{fh}$, χ_*x*_, *M*, $N_{B}^{men}$, $E_{B}^{ip}$, and $E_{B^{\prime }}^{ip}$, rank top six in determining the formation energy, revealing the dominant role of the BX_6_ and B′X_6_ octahedrons in the chemical stabilities of A_2_BB′X_6_ perovskites. The heat of formation $H_{X}^{fh}$ and electronegativity χ_*x*_ of X‐site halogen atom play the key role in △*H* ^*ML*^, which is consistent with the widely‐accepted point that the structural stability of HOIDPs is roughly affected by the geometric criteria *T*_*f*_ and *B* − *X* bonding strength,^[^ ^60^ ^]^ and the latter is usually determined by the difference of electronegativity and distance between bond partners. The X‐resolved dependence of △*H* ^*ML*^ on *M* are shown in Figure 2c, which reveals that △*H* ^*ML*^ of X‐based HOIDPs possess the relationship of $▵H_{F}^{ML}<▵H_{Cl}^{ML}<▵H_{Br}^{ML}<▵H_{I}^{ML}$, confirming that stronger electronegativity of halogen atom at X site or lighter molar mass *M* leads to lower formation energy. Based on the model of formation energy, we can obtain the formation energy of each candidate and reduce the number of candidates to 17 051, by excluding compounds with formation energies larger than ‐0.2 eV atom^‐1^.

#### 2.4.2 Electronic and Optical Properties: Band Gap

**Figure 3:**
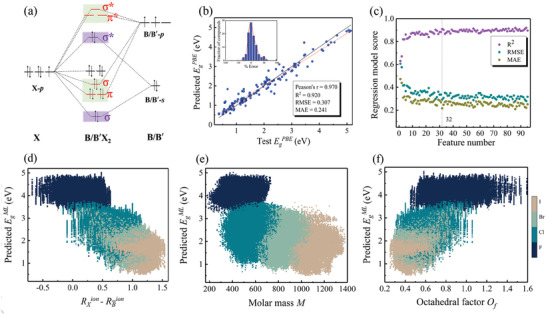
a) Schematic illustration of the band diagram for HOIDPs. The results of GBR model for band gap $E_{g}^{PBE}$ b) the actual value of band gap $E_{g}^{PBE}$ by DFT in the test set and the predicted value $E_{g}^{PBE}$ by ML. Pearson coefficient (r), coefficient of determination (R^2^), mean squared error (MSE), and mean absolute error (MAE) are calculated to explain the training performance of the built model. The ideal line is shown as red line and the fit line is shown as dark line. The inset is the fraction of compounds according to their percent error between predicted $E_{g}^{ML}$ and actual $E_{g}^{DFT}$. c) The values of R^2^, MSE, and MAE of GBR model with the number of selected features. Relationship visualization of the prediction of band gap for all electrically neutral HOIDP candidates with d) the difference of ion radius between X and B/B′ sites $R_{X}^{ion}-R_{\bar{B}}^{ion}$, e) molar mass *M*, and f) octahedral factor *O*_*f*_, respectively. Different colors represents different X‐site halogen elements.

The electronic and optical properties of semiconducting perovskite solar cells are fundamental to understand or further manipulate their interactions with light, and the production, transport, collection, and recombination of photo‐generated non‐equilibrium carriers. For large absorption coefficients the HOIDPs are required to possess direct band gap, since the optical transition of electrons for indirect‐bandgap semiconductors inevitably involves phonons to meet the conservation of momentums, which leads to very weak absorption. Furthermore, the *p* → *p* transition for electrons from valence band (VB) to conduction band (CB) in direct‐bandgap semiconductors is highly beneficial for large absorption coefficient. As we know now, the VB of HOIP, for example, CH_3_NH_3_PbX_3_ (*X* = *F*/*Cl*/*Br*/*I*), is usually formed by the antibonding states of *X* − *p* and *B*/*B*′ − *s* atomic orbitals, while the CB is formed by the antibonding states of *X* − *p* and *B*/*B*′ − *p* atomic orbitals,^[^ ^61^ ^]^ as shown in **Figure** **3**a. Since both VB and CB possess *p* −orbitals, the absorption coefficients for directly semiconducting HOIPs are generally large, beneficial for large PCE.

Here, we only consider HOIDPs with direct band gap. The GBR model for PBE band gap shown in Figure 3b reveals a good match between the predicted band gap and PBE‐calculated ones in the test set. By using GBR model to record the feature importance and combining “last‐place elimination” algorithm,^[^ ^35^ ^]^ the feature selection based on importance is achieved, as shown in Figure 3c. Each lavender dot is R^2^ value of GBR model during each selection process, each cyan dot represents MSE value, and each khaki dot denotes MAE value. Obviously, R^2^ abruptly decreases, and MSE and MAE abruptly increase when the number of feature is smaller than 32. So we select these 32 features as important features for PBE band‐gap model, as shown in Figure S3, Supporting Information.

The top 10 important features are $R_{X}^{ion}-R_{\bar{B}}^{ion}$, χ_*B*_, $\chi_{B^{\prime }}$, $R_{X}^{ion}/R_{\bar{B}}^{ion}(=1/O_{f}$), *M*_*A*_, *M*, *O*_*f*_, $N_{X}^{proton}$, *N*_*A*_, and *P*_*A*_, which manifest the key role of B/B′‐ and X‐sites in the formation of band gap, consistent with the aforementioned discussions. Since the VB and CB of HOIDPs are dominantly attributed to the atomic orbitals of B/B′ and X atoms, large energy‐level differences Δ*E* between B/B′ − *s*/*p* orbitals and X−*s*/*p* orbitals lead to large band gap, as shown in Figure 3a, Supporting Information. The *s*/*p* orbital energies for the halogen elements of *F*/*Cl*/*Br*/*I* can be calculated by the Rydberg model, that is, $E=\frac{-Z_{eff}^{2}}{n^{2}}13.6eV$, with *Z*_*eff*_ and *n* representing the effective nuclear charge and principal quantum number. The values of the orbital exponents ζ for neutral halogen atoms, defined as ζ = *Z*_*eff*_/*n*, are obtained as listed in Table S2, Supporting Information, then the *s*/*p* orbital energies can be calculated as $E_{Cl}^{3p}=-56.52eV>E_{Br}^{4p}=-69.28eV>E_{I}^{5p}=-73.33eV>E_{Cl}^{3s}=-75.49eV>E_{F}^{2p}=-88.43eV>E_{F}^{2s}=-89.41eV>E_{Br}^{4s}=-94.66eV>E_{I}^{5s}=-97.74eV$. When *F* → *Cl* → *Br* → *I*, $R_{X}^{ion}$ and $N_{X}^{proton}$ increase, as well as the absolute values of the energy‐level $E_{X}^{s/p}$ of X atoms. When the Mendeleev's number of B/B′ atoms $N_{B/B^{\prime }}^{men}$ decreases, generally $R_{B}^{ion}$ decreases and $\chi_{B/B^{\prime }}$ increases, and the absolute values of energy $E_{B/B^{\prime }}^{s/p}$ of B/B′ − *s*/*p* orbitals decrease. The X‐resolved dependences of band gap on $R_{X}^{ion}-R_{\bar{B}}^{ion}$ and *M* are shown in Figure 3d,e, respectively, which reveal that, as *F* → *Cl* → *Br* → *I*, $R_{X}^{ion}-R_{\bar{B}}^{ion}$ and *M* increase, and the band gap reduces, resulted from the decrease of energy difference Δ*E* between B/B′ − *s*/*p* and X−*s*/*p* atomic orbitals. The X‐resolved dependence of band gap on octahedral factor *O*_*f*_ is shown in Figure 3f, which reveals that, as *O*_*f*_ increases, indicating enhanced octahederal distortions in HOIDPs, band gap increase, which is due to the reduction of overlap between B/B′ − *s*/*p* orbitals and X−*s*/*p* orbitals by enhanced octahederal distortion, responsible for widening band gap. Similar analysis can be conducted on the influences from χ_*B*_ and $\chi_{B^{\prime }}$.

To further analyze the correlation among the selected 32 features, the Pearson correlation coefficient is calculated, which can produce the positive and negative correlation between one pair of features, and the results are shown in the inset of Figure S3, Supporting Information. If the correlation value between two features exceeds 0.8, the features with lower importance will be deleted in order to further reduce the redundancy of features. As shown in Figure S4, Supporting Information, through feature pruning process, the total number of features is decreased to 14 and the top 10 important features before pruning is decreased to 6 ($R_{X}^{ion}-R_{\bar{B}}^{ion}$, χ_*B*_, $\chi_{B^{\prime }}$, *M*_*A*_, *M*, *O*_*f*_).

In addition, as mentioned above, PBE calculations always underestimate the band gap for semiconductors, therefore, we also select HSE‐calculation results to obtain precise band gap for semiconducting HOIDPs and build ML model for HSE band gap. The related prediction performance is shown in Supporting Information, and the calculated R^2^, MSE, and MAE are 0.870, 0.384, 0.278, respectively, as shown in Figure S5a, Supporting Information. Compared with $E_{g}^{PBE}$ model, the top ten important features for ML‐models of HSE‐bandgaps are *M*, $R_{X}^{ion}-R_{\bar{B}}^{ion}$, the Mendeleev′s number of B${}^{\prime }$ and B, that is, $N_{B^{\prime }}^{men}$ and $N_{B}^{men}$, $R_{X}^{ion}+R_{\bar{B}}^{ion}$, $\chi_{B^{\prime }}$, χ_*B*_, $R_{X}^{ion}/R_{\bar{B}}^{ion}$, *P*_*A*_ and *O*_*f*_ as shown in Figure S5b, Supporting Information, which are nearly identical with those for $E_{g}^{PBE}$ models.

We apply these ML‐bandgap models to predict the band gap of 17051 HOIDP candidates screened on basis of formation energy, and 1183 candidates with band gap ranging from 0.6 to 2.2 eV and from 1.1 to 3.0 eV, respectively, for $E_{g}^{PBE}$ and $E_{g}^{HSE}$ beneficial for light harvesting and PCE of PSCs are generated.

#### 2.4.3 Thermal Properties: Debye Temperature

Although currently CH_3_NH_3_PbX_3_‐based PSCs are extensively used for photovoltaics, they not only suffer from the structural instabilities in ambient environment and toxicity due to lead, but also suffer from low lattice thermal conductivity κ_*L*_ due to low Debye temperatures Θ_*D*_ around 200 K,^[^ ^62^ ^]^ which may lead to decomposition of the crystals due to heat accumulation. Materials with low Θ_*D*_ tend to have low‐energy phonon modes, and enhance phonon‐phonon scattering processes since over Θ_*D*_ all phonons are excited, leading to the decrease of κ_*L*_ according to Slack model, and the increase of the probability of non‐radiative relaxation time, the latter of which directly decreases PCEs of PSCs. Therefore, the materials with high Θ_*D*_ are highly expected for the design of high‐efficiency and highly stable PSCs. According to the formulae for Θ_*D*_ shown as Equation (15), Θ_*D*_ value is related to the bond strength in crystals.

Based on the high‐throughput calculation of Debye temperatures for 425 HOIDPs, GBR model predicts $\Theta_{D}^{ML}$ well matched with the calculated $\Theta_{D}^{DFT}$ as shown in Figure S7a, Supporting Information. Based on the feature‐importance selection process as shown in Figure S6b, Supporting Information, 28 important features are selected as shown in Figure S7b, Supporting Information. The top ten important features are χ_*X*_, $R_{X}^{ion}+R_{\bar{B}}^{ion}$, $E_{X}^{ip}$, *M*, $R_{X}^{ion}$, *T*_*f*_, $N_{X}^{proton}$, $E_{X}^{pa}$, $N_{X}^{period}$, and $R_{X}^{s+p}$, which manifest the important role of corner‐sharing B/B′X_6_ octahedra in determining Θ_*D*_, and five of them are sharing with those for the ML model of formation energy. We also calculate the Pearson correlation value between Debye temperatures and formation energies, and the calculated result is 0.42, indicating the high correlation between Θ_*D*_ and Δ*H*, which is due to the similar underlying mechanism of *B*/*B*′ − *X* bond strength for Θ_*D*_ and Δ*H*.

The X‐resolved dependence of Θ_*D*_ on χ_*X*_, *M*, *T*_*f*_, and *O*_*f*_ is shown in Figure S8a– d, Supporting Information, respectively. As shown in Figure S8a,b, Supporting Information, as *F* → *Cl* → *Br* → *I*, the value of χ_*X*_ decreases, and as a result *B*/*B*′ − *X* bonds weaken, as well *M* increases, leading to the decrease of Θ_*D*_. Similar tendency regarding X‐site contribution to Θ_*D*_ can be observed in Figure S8c,d, Supporting Information. Different from those in case of Δ*H*, the Goldsmith tolerance factor *T*_*f*_, which indicates the deviation of HOIDP structures from cubic crystal, also plays an important role in determining Θ_*D*_, as shown in Figure S8c, Supporting Information, which reveals that, as *T*_*f*_ increases, Θ_*D*_ increases. According to the ML model for Θ_*D*_, we select 597 HOIDPs from 1183 candidates, whose Debye temperature is higher than 500 K (much larger than Θ_*D*_ of CH_3_NH_3_PbX_3_ crystal).

## 3 Discussions

It is worth to mention that three compounds ((CH_3_NH_3_)_2_AgSbI_6_, (CH_3_NH_3_)_2_AgBiBr_6_, and (CH_3_NH_3_)_2_TlBiBr_6_) from 1183 HOIDP candidates screened from the criteria of formation energy and band gap have been experimentally synthesized, and the predicted HSE‐bandgaps for them are quite close to the experimentally observed ones, as shown in Table S3, Supporting Information, validating the precision of our ML models. After screening the HOIDP candidates with the chemical space of 180 038 by formation energy less than ‐0.2 eV atom^‐1^, PBE/HSE band gap ranging from 0.6/1.1 to 2.2/3.0 eV and Debye temperatures larger than 500 K, 597 HOIDP candidates are subsequently selected, which are probable to possess chemical stability, appropriate band gap and good thermal transport property. Furthermore, considering selecting non‐toxic elements and accessible Br‐based HOIDPs in experiments,^[^ ^63^ ^]^ 12 rhombohedral Br‐based HOIDPs are finally selected for further detailed investigations on their electronic, optical, photovoltaic, and thermal properties by DFT calculation.

Since different elements occupy the vertices of the regular corner‐sharing BX_6_ and B′X_6_ octahedra and the lone‐pair electrons of the B/B′‐site ions leads to the coordination symmetry breaking, all these 12 undetected HOIDPs have different degrees of distorted BX_6_/B′X_6_ octahedra, although they possess typical perovskite structures as listed in Table S4, Supporting Information. The calculated octahedral factors *O*_*f*_ for these 12 HOIDP candidates are listed in **Table** **1**, which reveals a larger *O*_*f*_ for Ag‐based HOIDPs compared to Ti‐based HOIDPs. The calculated formation energies for them are smaller than ‐0.35 eV  atom^‐1^, most of which are well smaller than those of CH_3_NH_3_PbI_3_ (‐0.39 eV  atom^‐1^^[^ ^32^ ^]^), confirming their chemical stabilities.

**Table 1:** Twelve selected HOIDPs with relevant properties, where *c* and *p* represent calculated and predicted results, respectively, and $p-E_{g}^{PBE1}$ and $E_{g}^{PBE2}$ represent the predicted results from the PBE band gap models of 4456 and 425 HOIDPs, respectively

Formula	*T*_*f*_	*O*_*f*_	*c* − Δ*H*	*c* − Θ_*D*_	$c-E_{g}^{PBE}$/$E_{g}^{HSE}$	*c* − κ_*L*_	Direct/Indirect	*p* − Δ*H*	*p* − Θ_*D*_	$p-E_{g}^{PBE1}$/$E_{g}^{PBE2}$ /$E_{g}^{HSE}$
(CH_3_NH_3_)_2_AgAlBr_6_	1.02	0.54	‐0.42	544.9	1.57 / 2.94	5.49	Direct	‐0.41	525.3	0.67 / 1.73 / 2.99
(CH_3_NH_3_)_2_AgGaBr_6_	1.00	0.56	‐0.37	518.3	0.93 / 1.97	5.04	indirect	‐0.36	511.0	0.86 / 1.60 / 2.48
(CH_3_NH_3_)_2_AgInBr_6_	0.97	0.61	‐0.38	496.9	0.51 / 1.70	4.39	Direct	‐0.36	502.5	0.43 / 0.73 / 2.12
(HC(NH_2_)_2_)_2_AgGaBr_6_	1.11	0.56	‐0.45	521.4	2.35 / 3.84	4.63	Direct	‐0.40	525.6	0.65 / 2.59 / 2.94
(C_2_NH_6_)_2_AgTiBr_6_	1.02	0.56	‐0.49	572.9	0.00 /2.49	5.73	Indirect	‐0.43	554.9	0.93 / 1.82 / 2.52
(C_2_NH_6_)_2_AgAlBr_6_	1.03	0.54	‐0.47	577.4	1.85 / 3.29	5.74	Indirect	‐0.39	555.3	1.61 / 1.78 / 2.97
(C_2_NH_6_)_2_AgInBr_6_	0.99	0.61	‐0.44	535.5	1.13 / 2.48	5.16	Indirect	‐0.31	515.0	1.24 / 1.60 / 2.47
(C_2_OH_5_)_2_AgAlBr_6_	1.04	0.54	‐0.99	545.1	1.86 / 3.20	5.37	Indirect	‐0.70	523.7	1.42 / 1.68 / 2.93
(C_2_NH_6_)_2_TiTiBr_6_	1.13	0.41	‐0.75	628.6	0.00 / 1.67	6.96	Indirect	‐0.62	591.2	0.93 / 1.80 / 2.01
(C_2_NH_6_)_2_TiMnBr_6_	1.12	0.43	‐0.35	618.4	0.00 / 2.62	6.53	Indirect	‐0.31	586.6	0.93 / 1.78 / 2.40
(C_2_NH_6_)_2_TiZnBr_6_	1.10	0.45	‐0.48	581.2	0.00 / 1.70	5.77	Indirect	‐0.41	571.6	0.85 / 1.59 / 1.94
(C_2_NH_6_)_2_TiGeBr_6_	1.15	0.39	‐0.45	578.9	0.00 / 1.02	5.59	Indirect	‐0.27	560.8	0.82 / 1.57 / 1.87

**Figure 4:**
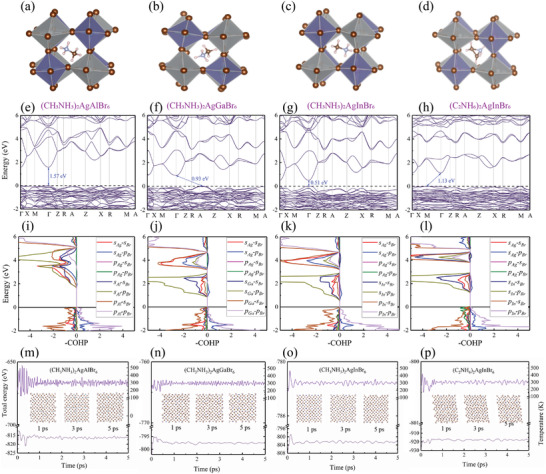
a–d) DFT optimized crystal structure, e–h) the calculated electronic band structures, i–l) orbital‐resolved‐pCOHP, m–p) the variation of total energy and crystal structure during 5 ps AIMD simulations at room temperature for selected four HOIDPs (CH_3_NH_3_)_2_AgAlBr_6_, (CH_3_NH_3_)_2_AgGaBr_6_, (CH_3_NH_3_)_2_AgInBr_6_, and (C_2_NH_6_)_2_AgInBr_6_, respectively.

The PBE/HSE calculations of band gap for these 12 HOIDP candidates are listed in Table 1 as well, which are in good agreement with ML‐predicted ones for Ag‐based HOIDP candidates, but PBE calculations give zero band gap for Ti‐based HOIDP candidates. However, HSE calculations give band gap well matched with those predicted by HSE ML‐model for all these 12 HOIDPs. By using the criterion of HSE band gap ranging from 1.1 to 3.0 eV and excluding Ti‐based HOIDPs, four HOIDPs of (CH_3_NH_3_)_2_AgAlBr_6_, (CH_3_NH_3_)_2_AgGaBr_6_, (CH_3_NH_3_)_2_AgInBr_6_, and (C_2_NH_6_)_2_AgInBr_6_ are selected in the end as shown in **Figure** **4**a–d, respectively. The PBE‐calculated bandstructures for these four HOIDPs are shown in Figure 4e–h, respectively, which reveals that, (CH_3_NH_3_)_2_AgAlBr_6_ and (CH_3_NH_3_)_2_AgInBr_6_ possess direct band gap, and the HSE‐calculations of band gap give 2.94 and 1.70 eV, respectively, well consistent with ML‐predicted ones as listed in Table 1. Since the difference of the band gap for these two HOIDPs is large, therefore the band gap for (CH_3_NH_3_)_2_AgAl_*x*_In_1 − *x*_Br_6_ can be tunable by manipulating the Al ratio according to the bowing effect.^[^ ^64^ ^]^ In addition, the hole or electron effective mass of these 12 HOIDP candidates in *x*/*y*/*z* direction are different as listed in Table S5, Supporting Information, and the average of hole effective mass is usually higher electron's except (CH_3_NH_3_)_2_AgGaBr_6_, (C_2_NH_6_)_2_TiTiBr_6_, and (C_2_NH_6_)_2_TiZnBr_6_, which indicates the hole extraction in transport layer is more difficult and employing excellent hole transport layer will lead to high photovoltaic performance in the actual PSC design.

To further reveal the chemical bonding states for VB and CB in (CH_3_NH_3_)_2_AgAlBr_6_, (CH_3_NH_3_)_2_AgGaBr_6_, (CH_3_NH_3_)_2_AgInBr_6_, and (C_2_NH_6_)_2_AgInBr_6_, we also use the crystal orbital Hamilton population (COHP) method proposed by Bloechl et al.^[^ ^65^ ^]^ to calculate their Hamiltonian elements with positive/negative values indicating the bonding/antibonding states between chosen atomic orbitals. The calculated orbital‐resolved ‐pCOHP for these four HOIDPs are shown in Figure 4i–l, which reveals that the CBs in all these HOIDPs are dominantly formed by the antibonding states of *B*′ − *s* and *Br* − *p*, *B*′ − *s* and *Br* − *s*, and *B* − *s* and *Br* − *s*, with *B* = *Ag* and *B*′ = *Al*/*Ga*/*In*, and the VBs for these four HOIDPs are dominantly formed by the antibonding states of *B*′ − *p* and *Br* − *s*. Similar to the case in CH_3_NH_3_PbI_3_ mentioned above, the absorption coefficients of these four HOIDPs will be beneficial from the *p* orbitals involved in their CBs and VBs. In addition, we also calculate the electron localization function (ELF), which is position‐dependent with the value ranging from 0.0 to 1.0. The value of unity for ELF reflects maximum probability to find the localized electrons, ELF = 0.5 corresponds to the electron‐gas‐like pair behavior for electrons, and the value less than 0.5 of ELF means electrons are emptied from this position. The calculated ELFs for these four HOIDPs are shown in Figure S9, Supporting Information, which reveals that electrons are emptied from the B/B′ metal‐atom sites and accumulated at the X halogen‐atom sites, indicating that *B*/*B*′ − *X* bonds are essentially ionic.

We also conduct AIMD simulations to investigate the thermal stability of these four HOIDPs at 300 K for the total simulation time of 5 ps with a time interval of 1 fs, as shown in Figure 4m–p, which reveal that during simulation time, their time‐dependent total energies fluctuate in a narrow range and the crystal structures remain integrated, indicating their thermal stabilities at 300 K. Furthermore, for the results of time‐dependent evolution of XRD simulations, as shown in Figure S10, Supporting Information, no shift diffraction peaks appear, which further confirms that these selected HOIDPs have no change in structure and keep their structural integrity in the process of simulations. In addition, we calculate the general tolerance factor proposed by Filip et al.^[^ ^66^ ^]^ for these four HOIDPs to check the limits of stretch, octahedral, tilt, and chemistry shown in Table S6, Supporting Information, which indicates that (CH_3_NH_3_)_2_AgGaBr_6_, (CH_3_NH_3_)_2_AgInBr_6_, and (C_2_NH_6_)_2_AgInBr_6_ meet the stability criteria for double perovskites. Furthermore, the H_2_O adsorption energy of four selected HOIDPs estimated by $E_{ads}=E_{slab+H_{2}O}-E_{slab}-E_{H_{2}O}$, with $E_{slab+H_{2}O}$, *E*_*slab*_, and $E_{H_{2}O}$ the total energies of H_2_O adsorbed perovskite slab, perovskite slab, and the isolated H_2_O molecule, respectively, is calculated to check the stabilities against H_2_O. The H_2_O molecule is adsorbed along the z‐direction of perovskite slab with the vacuum thickness of 18 $\overset{\circ }{A}$. Comparing with CH_3_NH_3_PbI_3_ whose H_2_O‐adsorption energy is ‐0.48 eV, (CH_3_NH_3_)_2_AgAlBr_6_, (CH_3_NH_3_)_2_AgGaBr_6_, and (CH_3_NH_3_)_2_AgInBr_6_ show better environmental stability against H_2_O with *E*_*ads*_ of ‐0.32, ‐0.42, and ‐0.04 eV, respectively. H_2_O‐adsorption energy for (C_2_NH_6_)_2_AgInBr_6_ is ‐1.87 eV.

For accurate calculation of device performance of PSCs, for example, PCE, we also calculate the quasi‐particles (QP) band structures for excited states based on many‐body perturbation theory (MBPT) and obtain the optical absorption by considering many‐body electron–hole interactions. We use one‐shot G_0_W_0_ method to calculate the QP states based on the calculated Kohn–Sham (KS) orbitals implemented in Quantum Espresso software package,^[^ ^67^, ^68^ ^]^ and on top of QP states, the electron–hole excitation states for these four HOIDPs can be calculated using the G_0_W_0_+BSE method combined with the Tamm–Dancoff approximation method, which are implemented in Yambo.^[^ ^69^ ^]^ By the summation of respective contribution from a large number of electron‐hole pairs according to Equation (9), the imaginary part of the dielectric constant ϵ_2_ can be obtained, and the absorption can be subsequently calculated as well.

**Figure 5:**
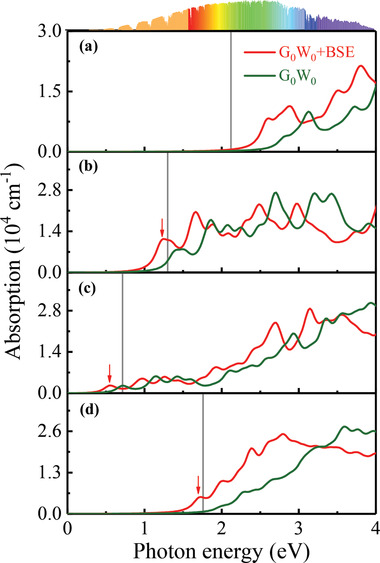
Energy‐dependent optical absorption calculated by G_0_W_0_+BSE of selected four HOIDPs a) (CH_3_NH_3_)_2_AgAlBr_6_, b) (CH_3_NH_3_)_2_AgGaBr_6_, c) (CH_3_NH_3_)_2_AgInBr_6_, and d) (C_2_NH_6_)_2_AgInBr_6_. The gray line indicates the QP bandgap.

The calculated QP band gap for (CH_3_NH_3_)_2_AgAlBr_6_, (CH_3_NH_3_)_2_AgGaBr_6_, (CH_3_NH_3_)_2_AgInBr_6_, and (C_2_NH_6_)_2_AgInBr_6_ are 2.12, 1.30, 0.71, and 1.76 eV, respectively. As shown in **Figure** **5**, the calculated absorption with and without considerations of electron–hole interactions for the selected four HOIDPs, are plotted by red and green lines, respectively. Obviously, the contribution from electron–hole interactions influences the optical absorption significantly, and no excitonic absorption peak can be observed in (CH_3_NH_3_)_2_AgAlBr_6_ crystal, as shown in Figure 5a. The absorption spectra for (CH_3_NH_3_)_2_AgAlBr_6_, (CH_3_NH_3_)_2_AgGaBr_6_, (CH_3_NH_3_)_2_AgInBr_6_, and (C_2_NH_6_)_2_AgInBr_6_ cover the ultrabroadband region ranging from 180/163/180/143 nm to 496/1127/2480/775 nm, respectively. Notably, the absorption spectra for all the four HOIDPs cover the major part of the solar‐energy spectrum as shown in Figure 5, especially for (CH_3_NH_3_)_2_AgGaBr_6_ and (C_2_NH_6_)_2_AgInBr_6_, indicating their abilities to efficiently harvesting the solar energy by photovoltaic effects similar to CH_3_NH_3_PbI_3_. The excitonic peaks within the QP bandgap for (CH_3_NH_3_)_2_AgGaBr_6_, (CH_3_NH_3_)_2_AgInBr_6_, and (C_2_NH_6_)_2_AgInBr_6_ locate at 1.22, 0.54, 1.70 eV, respectively, denoted by red arrows as shown in Figure 5, and the corresponding excitonic binding energies defined as the energy difference between the excitonic eigenenergy and QP bandgap, are 0.08, 0.17, and 0.06 eV, respectively, which are comparable to 0.05 eV for CH_3_NH_3_PbI_3_ crystal.^[^ ^70^ ^]^ Among them, the relatively large binding energy of 0.17 eV in (CH_3_NH_3_)_2_AgInBr_6_ is observed, implying the potential applications for light‐emission devices. The vanishing excitonic absorption peaks or small excitonic binding energies for these four HOIDPs imply easy dissociation of photogenerated excitons, which is beneficial for high‐performance PSC devices. The calculated energy‐dependent optical absorption of CH_3_NH_3_PbI_3_ is shown in Figure S12, Supporting Information, for comparison. In addition, we also conduct the analysis on the selection rule for optical transition based on the group‐theory argument for four HOIDP candidates which is provided in Tables S7‐S10, Supporting Information. Actually, for the four candidates, the transitions of valence‐band electrons at some high‐symmetric points in the Brillouin zone are allowed, and some are forbidden, which means that, not all the optical transition between valence bands and conduction bands are forbidden restricted by the parity‐induced dipole‐forbidden rule. Although the optical transition between VBM and CBM is forbidden in some double perovskites, those transitions around the band edge are still allowed due to the *C*_1_ symmetry, thus the optical properties can be still significantly large.

As we know, the creation of the tail states in the band edge of materials generates serious increase of open‐circuit voltage losses *V*_*loss*_, which can be characterized quantitatively through the evaluation of absorption tail, expressed by $\alpha =\alpha_{0}+e^{\left(\frac{E}{E_{u}}\right)}$, where α is the absorption coefficient, *E* is the photon energy, and *E*_*u*_ is the Urbach energy.^[^ ^71^ ^]^ The Urbach energy is presumed as the width of the tail of localized defect states in the band gap. It has already been reported that *E*_*u*_ shows a direct correlation with *V*_*loss*_ and the smaller *E*_*u*_ (i.e., sharper absorption edge) is beneficial to suppressing *V*_*loss*_. Based on the absorption curves calculated by the precise G_0_W_0_+BSE method for the four selected candidates, we calculate their Urbach energies *E*_*u*_ by fitting the reciprocal of the slope of *ln*(α), which gives 58.0, 18.3, 18.1, and 28.9 meV for (CH_3_NH_3_)_2_AgAlBr_6_, (CH_3_NH_3_)_2_AgGaBr_6_, (CH_3_NH_3_)_2_AgInBr_6_, and (C_2_NH_6_)_2_AgInBr_6_, respectively, as shown in Figure S13, Supporting Information, in comparison to the case for CH_3_NH_3_PbI_3_ as shown in Figure S14, Supporting Information, giving *E*_*u*_ of 21.9 meV consistent with the experimental result of 11.3 meV.^[^ ^72^ ^]^

**Figure 6:**
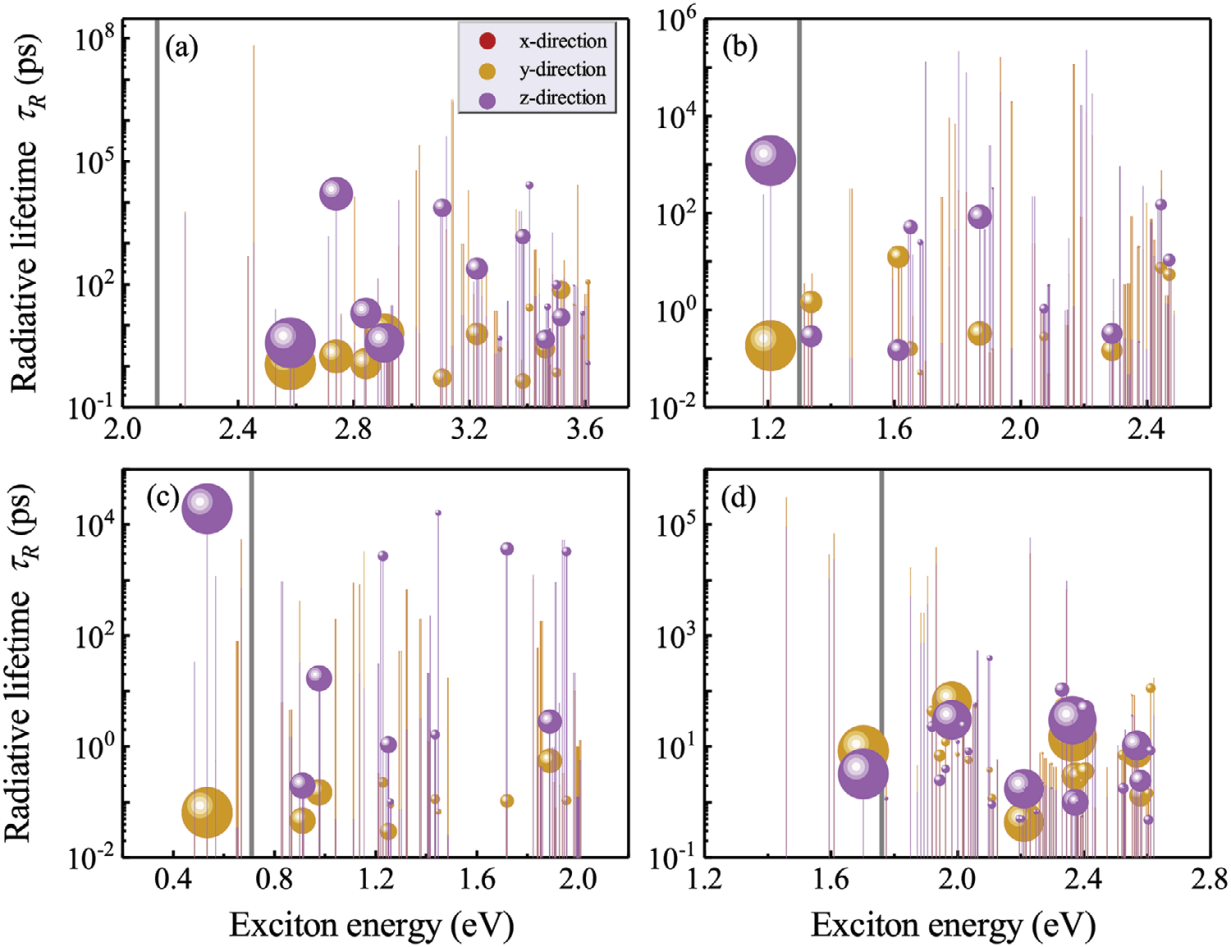
Exciton lifetimes induced by many‐body interactions for selected HOIDPs. The gray line is QP band gap and the circle radius shows the contribution from exciton to excitonic absorption peak.

The calculated exciton‐resolved radiative combination rates τ_*R*_ at room temperature according to Equation (12) for these four HOIDPs are shown in **Figure** **6**, in which the circle radius is proportional to the oscillator strength for the corresponding exciton. The stronger oscillator strength leads to larger contribution from the exciton to the excitonic absorption peak. As shown in Figure 6, τ_*R*_ is isotopic irrespective of *x*‐ or *y*‐polarization but z‐polarization is different, and the τ_*R*_ values for excitons with large oscillator strength range from 10 ps to 10 ns, comparable to 14 ns for CH_3_NH_3_PbI_3_ and 37 ns for CH_3_NH_3_PbBr_3_.^[^ ^73^ ^]^

According to Equation (20), the calculated short‐circuit currents *J*_*sc*_ for (CH_3_NH_3_)_2_AgAlBr_6_, (CH_3_NH_3_)_2_AgGaBr_6_, (CH_3_NH_3_)_2_AgInBr_6_, and (C_2_NH_6_)_2_AgInBr_6_ are 7.0, 23.4, 53.2, 20.8 mA*cm* ^−2^. Then, according to Equation (18), the calculated PCEs for these four HOIDPs are 11.4%, 20.6%, 19.9%, and 27.6%, respectively. The relatively smaller PCE for (CH_3_NH_3_)_2_AgAlBr_6_ compared to the rest is probable due to the smaller overlap of the absorption spectrum and solar‐energy spectrum as shown in Figure 5a.

In hybrid perovskites, since the materials are generally soft and polar, charged carriers are generally dressed in phonons resulted from large dielectric electron‐phonon couplings, forming the so‐called polarons. Recent experimental and theoretical studies have shown that the coupling between polar longitudinal optical (LO) phonon and carriers, that is, the Fröhlich interaction, dominates the charge carrier dynamics in hybrid perovskites at room temperatures, and the low‐frequency LO phonon vibrations, namely, X‐B‐X bond bending and stretching motions, contribute dominantly to the Fröhlich interaction.^[^ ^74^ ^]^ The Fröhlich coupling strength is defined as, $\alpha_{F}=\frac{e^{2}}{\hbar }\left(\frac{1}{\varepsilon_{\infty }}-\frac{1}{\varepsilon_{0}}\right)\sqrt{\frac{m}{2\hbar w}}$ where ε_∞_ and ε_0_ are the high‐frequency and static dielectric constant, and ω is the LO‐phonon frequency. In the weak coupling regime, that is, α_*F*_ ⩽ 2, large polarons form. We calculate the electron and hole α_*F*_ for the four selected HOIDP candidates and the results are listed in Table S11, Supporting Information, which shows that, their hole α_*F*_ (3.35, 5.54, 2.80, and 2.71 for (CH_3_NH_3_)_2_AgAlBr_6_, (CH_3_NH_3_)_2_AgGaBr_6_, (CH_3_NH_3_)_2_AgInBr_6_, and (C_2_NH_6_)_2_AgInBr_6_, respectively) are larger than CH_3_NH_3_PbI_3_ of 2.67,^[^ ^75^ ^]^ and their electron α_*F*_ (1.48, 4.10, 2.32, and 1.60 for (CH_3_NH_3_)_2_AgAlBr_6_, (CH_3_NH_3_)_2_AgGaBr_6_, (CH_3_NH_3_)_2_AgInBr_6_, and (C_2_NH_6_)_2_AgInBr_6_, respectively) are comparable to CH_3_NH_3_PbI_3_ of 2.39,^[^ ^75^ ^]^ revealing the formation and existence of large or intermediate polarons in the four selected HOIDP candidates, similar to CH_3_NH_3_PbI_3_. To further investigate polaron behaviors in the predicted HOIDP candidates, we also calculate their polaron masses and polaron radii, and the results are listed in Table S11, Supporting Information.

Furthermore, we calculate the polaron mobility by solving the low‐field temperature‐dependent mobility equation with the FHIP, Kadanoff and Hellwarth methods, implemented in the *PolaronMobility.jl* code developed by J. M. Frost.^[^ ^76^ ^]^ The results are listed in Table S11, Supporting Information, which reveal that the electron polaron mobilities for (CH_3_NH_3_)_2_AgAlBr_6_, (CH_3_NH_3_)_2_AgGaBr_6_, (CH_3_NH_3_)_2_AgInBr_6_, and (C_2_NH_6_)_2_AgInBr_6_ are 90, 8, 53, 81 cm^2^V^−1^s^−1^, respectively, which are smaller than that of CH_3_NH_3_PbI_3_ of 197 cm^2^V^−1^s^−1^ but comparable to that of double‐perovskite Cs_2_AgBiBr_6_ of 34 cm^2^V^−1^s^−1^ calculated by the Kadanoff method.^[^ ^77^ ^]^ The hole polaron mobilities for (CH_3_NH_3_)_2_AgAlBr_6_, (CH_3_NH_3_)_2_AgGaBr_6_, (CH_3_NH_3_)_2_AgInBr_6_, and (C_2_NH_6_)_2_AgInBr_6_ are 5, 2, 27, 12 cm^2^V^−1^s^−1^, respectively, which are similarly smaller than that of CH_3_NH_3_PbI_3_ of 133 cm^2^V^−1^s^−1^, but comparable to that of double‐perovskite Cs_2_AgBiBr_6_ of 42 cm^2^V^−1^s^−1^.^[^ ^77^ ^]^ In addition, the carrier mobility induced by deformation potential scattering due to long‐wavelength acoustic phonons implemented by the ab initio scattering and transport AMSET code^[^ ^78^ ^]^ for the four HOIDP candidates is also calculated and the results are shown in Table S11, Supporting Information, which reveals that, in comparison to inorganic Cs_2_AgBiBr_6_, for the four selected candidates, the deformation potential scattering limited carrier mobilities are larger by two to three orders than those limited by Fröhlich coupling, especially for electrons. Therefore, the Fröhlich coupling dominates in the four selected HOIDP candidates.

As listed in Table 1, the calculated Debye temperatures for (CH_3_NH_3_)_2_AgAlBr_6_, (CH_3_NH_3_)_2_AgGaBr_6_, (CH_3_NH_3_)_2_AgInBr_6_, and (C_2_NH_6_)_2_AgInBr_6_ are 544.9, 518.3, 496.9, and 535.5 K, respectively, much larger than those in CH_3_NH_3_PbI_3_ crystal, which is probable due to the stiffer properties and larger phonon velocities of these four HOIDPs, since they have larger optical phonon frequencies resulted from smaller densities as listed in Table S5, Supporting Information. The corresponding calculated Grueneisen parameters γ are 1.28, 1.28, 1.33, and 1.31, respectively, which are larger than 1.17 experimentally reported in CH_3_NH_3_PbI_3_ crystal,^[^ ^62^ ^]^ implying enhanced anharmonic interactions among phonons in these four HOIDPs. According to the aforementioned Slack model, the calculated thermal conductivities κ_*L*_ at 300 K for (CH_3_NH_3_)_2_AgAlBr_6_, (CH_3_NH_3_)_2_AgGaBr_6_, (CH_3_NH_3_)_2_AgInBr_6_, and (C_2_NH_6_)_2_AgInBr_6_ are 5.49, 5.04, 4.39, and 5.16 *Wm* ^−1^ *K* ^−1^, which are much larger than experimentally reported 0.5 *Wm* ^−1^ *K* ^−1^ at room temperature in CH_3_NH_3_PbI_3_ crystal,^[^ ^20^ ^]^ indicating their good thermal transport properties beneficial for heat dissipation. Therefore, although the anharmonic interactions in these four HOIDPs are larger than CH_3_NH_3_PbI_3_ crystal, the much larger Debye temperature resulted from larger stiffness overrides the anharmonic interactions, leading to the larger thermal conductivities by an order in (CH_3_NH_3_)_2_AgAlBr_6_, (CH_3_NH_3_)_2_AgGaBr_6_, (CH_3_NH_3_)_2_AgInBr_6_, and (C_2_NH_6_)_2_AgInBr_6_.

## 4 Conclusion 

By combining high‐throughput DFT calculation with ML technology, we developed a multi‐step material screening scheme to accelerate the discovery of hybrid organic‐inorganic double perovskite material A_2_BB′X_6_. 597 stable HOIDP solar cell materials with suitable band gap and high Debye temperature were successfully selected from 180038 compounds. After that, based on the conditions of non‐toxicity and experimental accessibility, 12 candidate materials were selected, and four of them pass the verification of DFT with excellent properties. More importantly, with the help of ML technology, the prediction results of our HSE band gap model can achieve the level of results of experimental preparation, and the multi‐stage scheme of band gap prediction could ensure the validity of final candidates. The structure property relationship under different targets is established by full use of input data, which provides a further understanding of material properties. After DFT verification, the screened‐out candidates possess excellent properties.

As a new generation of material design strategy, ML driven scheme can achieve high‐precision material discovery in a “cheap” way without deep physical and chemical knowledge, just based on existing data and appropriate algorithms. Meanwhile, ML technology can capture the structure property relationship hidden in the data, which provides a unique way to understand the complex material properties, so it can help researchers jump out of the framework of known knowledge and find more suitable descriptors. In addition, multi‐objective screening can effectively improve the screening efficiency, and the number of targets is not limited. However, the limitations of ML‐based material design method also exist, such as the prediction accuracy depends on the reliability of dataset, which can be reflected from our two PBE band gap models. It is full of challenges to obtain the dataset with consistent data samples but also to ensure the diversity of samples. We choose the mandatory way that the predicted values of the two models both need to meet the requirement at the expense of the number of final candidates. A more flexible solution could be explored in the future research.

## 5 Methodology

### 5.1 Gradient Boosted Regression

Gradient boosted regression (GBR) is one of the tree‐based machine learning algorithms, which is based on the gradient boosting method and realized in the open‐source Scikit‐learn package.^[^ ^79^ ^]^ It is a single strong learner *f*(*x*) through combining many weak learners step by step using the gradient descent algorithm. It means that GBR usually find the gradient of *f*(*x*) which can minimize the average value of loss function to achieve the improvement of the accuracy of regression results. First, the model is initialized with a constant and the specific expression is as follows:

(1) $$f_{0}\left(x\right)=\underset{c}{argmin}\sum_{i=1}^{m}L\left(y_{i},c\right)$$

here *x* and *y*_*i*_ are the input and output values in the training set, *c* is the step size of gradient learning, and *m* is the number of data samples. Basic learner *h*_*j*_(*x*) needs to fit the negative gradient ${\tilde{y}}_{j}\left(x_{i}\right)$ of the loss function *L*(*y*_*i*_, *f*(*x*_*i*_)) for *n* (*j* < *n*) times

(2) $${\tilde{y}}_{j}\left(x_{i}\right)=-{\left[\frac{\partial L(y_{i},f\left(x_{i}\right))}{\partial f(x_{i})}\right]}_{f\left(x\right)=f_{j-1}\left(x\right)}$$

The next optimization function is solved to get each *c*, and the equation is as follows:

(3) $$c_{j}=\underset{c}{argmin}\sum_{i=1}^{m}L\left(y_{i},f_{j-1}\left(x_{i}\right)+ch_{j}\left(x_{i}\right)\right)$$

After that, the model is obtained with the following form:

(4) $$f_{j}\left(x\right)=f_{j-1}\left(x\right)+c_{j}h_{j}\left(x\right)$$

Therefore, the final model *f*_*n*_(*x*) is applied to predict.

### 5.2 Hyper‐Parameters Selection

In ML algorithm, in order to improve the efficiency and generalization performance of model, the hyper‐parameters of each ML algorithm should be selected before starting the formal training process. The optimization of hyper‐parameters is very important and this can be completed through a cross‐validated grid search or a randomized search over the parameter setting. For GBR algorithm, the related hyper‐parameters are loss function, learning rate, the number of boosting stages to perform, maximum depth of the individual regression estimators, the minimum number of samples required to be at a leaf node, and the number of features to consider when looking for the best split. When many hyper‐parameters exists together, the speed of traditional way of optimizing hyper‐parameters will be slow. A global search algorithm based on the simulated annealing algorithm is used to solve this problem, which is an open‐source python module called hyperopt.^[^ ^80^ ^]^ Through this module, the speed of hyper‐parameters search can be greatly improved. This search method is also applied to other algorithms involved in this article. The prediction results of our GBR model are the average values through ten cross‐validations after 100 iterations.

### 5.3 DFT Calculations

The calculations of material properties are performed using the Vienna ab initio simulation package (VASP), which is based on DFT.^[^ ^81^ ^]^ The pseudopotential used to describe the interaction between valence electrons and core charges is based on the projector‐augmented wave (PAW) method. We use the generalized gradient approximation (GGA) in parametrization of Perdew–Burke–Ernzerhof (PBE) version to describe the exchange‐correlation functional, with a kinetic energy cutoff of 600 eV. The K points in the Brillouin zone are sampled with the smallest k‐mesh interval of 0.2 under the Monkhorst‐Pack scheme. The convergence is set as 1 × 10^−5^ eV in total energy for two consecutive electronic steps and 0.01 eV Å^−1^ for maximum Hellman–Feynman force in the crystal. To eliminate the well‐known error in PBE calculation, we also use the Heyd–Scuseria–Erzenhof (HSE) hybrid functional method to improve the accuracy of the calculation of band gap for semiconductors. The hole and electron effective mass tensors, $m_{h}^{*}$ and $m_{e}^{*}$ are expressed as the double partial differential of the energy band E(k) at the valence band maximum (VBM) and conduction band minimum (CBM), respectively:^[^ ^82^ ^]^

(5) $$\frac{1}{m_{h,i,j}^{*}}=\frac{1}{\hbar^{2}}\frac{\partial^{2}E\left(k\right)}{\partial k_{i}\partial k_{j}}{|}_{k=VBM}\left(i,j=x,y,z\right)$$

(6) $$\frac{1}{m_{e,i,j}^{*}}=\frac{1}{\hbar^{2}}\frac{\partial^{2}E\left(k\right)}{\partial k_{i}\partial k_{j}}{|}_{k=CBM}$$

And the averages of diagonal terms in each tensor are used as the hole and electron effective mass, respectively.

In order to investigate the thermodynamic stability of candidates, we also carry out the ab initio molecular dynamics simulation (AIMD) in canonical ensemble to simulate the evolution of crystals at room temperature using the NOSè–Hoover method,^[^ ^83^ ^]^ where the NVT set is adopted and 2 × 1 × 2 supercell is constructed. A total of 5‐ps AIMD simulation is implemented with the time interval of 1 fs.

To investigate the optical properties of the candidates, we imply the G_0_W_0_+BSE method implemented by Yambo code to calculate the absorption spectra of those candidates.^[^ ^84^, ^85^ ^]^ The Kohn sham states are obtained using 5 × 5 × 5 Monkhorst‐Pack mesh on top of 200 empty bands. The quasiparticle (QP) states containing eight highest valance bands and eight lowest conduction bands are used in the Bethe–Salpeter equation kernel written as

(7) $$\left(\;E_{ck}^{QP}-E_{vk}^{QP}\right)A_{vck}^{s}+\sum_{v^{\prime }c^{\prime }k^{\prime }}\langle vck\left|K^{eh}\right|v^{\prime }c^{\prime }k^{\prime }\rangle A_{v^{\prime }c^{\prime }k^{\prime }}^{s}=\Omega_{S}A_{vck}^{S}$$

where $A_{vck}^{s}$ is the amplitude of the *S*th excitonic wave function, Ω_*S*_ is the corresponding excitonic eigenenergy, and *K* ^*eh*^ is the electron–hole interaction kernel. On the basis of the Tamm–Dancoff approximation, the exciton wave function in real space can be written as

(8) $$\Psi \left(r_{e},r_{h}\right)=\sum_{k,v,c}A_{vck}^{s}\psi_{k,c}\left(r_{e}\right)\psi_{k,v}^{*}\left(r_{h}\right)$$

The imaginary part of the dielectric function ϵ_2_ can be produced by summarization over all the exciton eigenstates,

(9) $$\varepsilon_{2}\left(\omega \right)=\frac{16\pi^{2}e^{2}}{\omega^{2}}\sum_{S}{\left|e\times \langle 0\left|v\right|S\rangle \right|}^{2}\delta \left(\omega -\Omega_{S}\right)$$

where *e* × 〈0|*v*|*S*〉, and *v* is velocity operator of the incident photons along polarization direction. The real part of the dielectric function ϵ_1_ can be obtained by the well‐known Kramers–Kronig relation as follows:

(10) $$\varepsilon_{1}\left(\omega \right)=1+\frac{2}{\pi }P\int_{0}^{\infty }\frac{\varepsilon_{2}\left(\omega^{\prime }\right)\omega^{\prime }}{\omega^{\prime 2}-\omega^{2}+i\eta }d\omega^{\prime }$$

Based on the calculated frequency‐dependent complex dielectric function, the absorption coefficient α(ω) can be calculated by as follows:

(11) $$\alpha \left(\omega \right)=\frac{\sqrt{2}\omega }{c}{\left\{{\left[\varepsilon_{1}^{2}\left(\omega \right)+\varepsilon_{2}^{2}\left(\omega \right)\right]}^{1/2}-\varepsilon_{1}\left(\omega \right)\right\}}^{1/2}$$

The radiative decay rate of a given exciton state *S* at temperature *T* for bulk crystal under the assumption that the exciton momentum has a thermal equilibrium distribution, can be calculated as follows:^[^ ^86^ ^]^

(12) $$\gamma_{S}\left(T\right)=\tau_{S}^{-1}=\frac{8\sqrt{\pi \varepsilon }e^{2}\hbar p_{S}^{2}}{3\varepsilon_{0}m^{2}VE_{S}{\left(0\right)}^{2}}{\left(\frac{E_{S}{\left(0\right)}^{2}}{2M_{S}c^{2}k_{B}T}\right)}^{3/2}$$

where the exciton energy *E*_*S*_(0) and the transition dipole $p_{S}$ (and $p_{S}^{2}={\left|p_{S}\right|}^{2}$) are produced by solving the BSE, and *M*_*S*_ denotes the exciton mass defined by $M_{S}=m_{e}^{*}+m_{h}^{*}$.

To investigate the coupling of carriers to acoustic phonons, we calculate the carrier mobility induced by deformation potential scattering implemented by the ab initio scattering and transport AMSET package, in which the electron‐acoustic phonon scattering matrix element is given by^[^ ^78^ ^]^

(13) $$g_{nm}^{ADP}=\langle mk+q|S_{q}:\left(D_{nk}+v_{nk}⨂v_{nk}\right)|nk\rangle$$

where |nk⟩ and |mk+q⟩ denote the initial and final states of electrons, respectively, $S_{q}$ is the strain associated with an acoustic phonon q, $v_{nk}$ is the group velocity for electron |nk⟩, and $D_{nk}$ is the second rank deformation potential tensor.

### 5.4 Formation Energy and Debye Temperature

For HOIDPs with the formulae of $A_{2}BB^{\prime }X_{6}$ under investigations here, the average formation energy per atom (△*H*) is defined as

(14) $$▵H=\left(E_{A_{2}BB^{\prime }X_{6}}-2E_{A}-E_{B}-E_{B^{\prime }}-3E_{X_{2}}-E_{H_{2}}\right)/N$$

where *N* is the number of atoms in the unit cell of HOIDPs compounds; $E_{A_{2}BB^{\prime }X_{6}}$, *E*_*A*_, *E*_*B*_, $E_{B^{\prime }}$, $E_{X_{2}}$, and $E_{H_{2}}$ are the total energies of HOIDPs unit cell, the isolated neutral organic molecule A, a single B atom and a single B′ atom in the corresponding elemental crystals, the isolated X_2_, and H_2_ molecules, respectively. For the group of tetramethylammonium cation (C_4_H_12_N^+^), the energy of neutral trimethylamine (C_3_H_9_N) is used for *E*_*A*_, and the energies of the molecules C_2_H_6_ is used instead of $E_{H_{2}}$.

To evaluate the thermal stabilities and thermal conductivities, we also investigate Debye temperature Θ_*D*_ for $A_{2}BB^{\prime }X_{6}$‐HOIDPs, which can be estimated on the basis of the results of the average sound velocity (*v*_*m*_), as follows:

(15) $$\Theta_{D}=\frac{h}{k_{B}}{\left[\frac{3n}{4\pi }\left(\frac{N_{A}\rho }{M}\right)\right]}^{\frac{1}{3}}v_{m}$$

where *h* is Planck constant, *k*_*B*_ is Boltzmann constant, *n* is the number of atoms per formula unit, *N*_*A*_ is Avogadro constant, ρ is the crystal structure's density, *M* is the molar mass, and *v*_*m*_ is the average sound velocity. In a poly‐crystalline material, *v*_*m*_ can be approximated as

(16) $$v_{m}={\left[\frac{1}{3}\left(\frac{2}{{v_{T}}^{3}}+\frac{1}{{v_{L}}^{3}}\right)\right]}^{-\frac{1}{3}}$$

here, *v*_*L*_ and *v*_*T*_ are the longitudinal and transverse sound velocity, respectively, which can be calculated from the bulk (*B*) and shear (*G*) moduli according to

(17) $$v_{L}={\left(\frac{B+\frac{4G}{3}}{\rho }\right)}^{\frac{1}{2}},v_{T}={\left(\frac{G}{\rho }\right)}^{\frac{1}{2}}$$

### 5.5 Device Performances for Photovoltaic PSCs

The key parameter to characterize the photovoltaic devices is PCE, also called as spectroscopic limited maximum efficiency, that, η,^[^ ^87^ ^]^ which is defined as the ratio of the maximum output power density (*P*_*max*_) and the total incident solar energy density (*P*_*in*_), as follows,

(18) $$\eta =\frac{P_{max}}{P_{in}}=\frac{max{\left\{(J_{sc}-J_{0}\left(e^{eV/k_{B}T}-1\right))V\right\}}_{V}}{\int_{0}^{\infty }EI_{sun}\left(E\right)dE}$$

where *P*_*max*_ is obtained by numerically maximizing the product of current density *J* and voltage *V*. We assume that the solar cell is illuminated under the photon flux *I*_*sun*_ and can be approximated as an ideal diode. At temperature *T*, the current density *J* and voltage *V* follow

(19) $$J=J_{sc}-J_{0}\left(e^{eV/k_{B}T}-1\right)$$

where *J*_*sc*_ is the short‐circuit current density, and

(20) $$J_{sc}=e\int_{0}^{\infty }\alpha \left(E\right)I_{sun}\left(E\right)dE$$

where α(*E*) is the energy‐dependent absorption, *e* is the elementary charge, and *I*_*sun*_ is the AM 1.5 G solar spectrum.^[^ ^88^ ^]^

The reverse saturation current *J*_0_ includes the radiative current $J_{0}^{r}$ and nonradiative current $J_{0}^{nr}$,

(21) $$J_{0}=J_{0}^{r}+J_{0}^{nr}=\frac{J_{0}^{r}}{f_{r}}$$

Here, $J_{0}^{r}=e\pi \int_{0}^{\infty }\alpha \left(E\right)I_{bb}\left(E,T\right)dE$ and the fraction of radiative recombination current *f*_*r*_ is given by $f_{r}=e^{\left(E_{g}-E_{g}^{da}/k_{B}T\right)}$, where *E*_*g*_ is the electronic band gap, $E_{g}^{da}$ is the direct‐allowed optical band gap, and *I*_*bb*_ is the black‐body spectrum at temperature *T*.

## Conflict of Interest

The authors declare no conflict of interest.

## Supporting information

Supporting Information

## Data Availability

The data that support the findings of this study are available from the corresponding author upon reasonable request.
