# Supplementary material for: Discovery of Lead‐Free Perovskites for High‐Performance Solar Cells via Machine Learning: Ultrabroadband Absorption, Low Radiative Combination, and Enhanced Thermal Conductivities
Source: Adv Sci (Weinh). 2021 Dec 14;9(4):2103648. doi: 10.1002/advs.202103648 (PMC8811845; doi:10.1002/advs.202103648)
Supplement: Supplementary file 1 — Supporting Information [file ADVS-9-2103648-s001.pdf]

ADVANCED SCIENCE
Open Access

Supporting Information

for *Adv. Sci.,* DOI: 10.1002/advs.202103648

Discovery of Lead-Free Perovskites for High-Performance Solar Cells via Machine Learning: Ultrabroadband Absorption, Low Radiative Combination, and Enhanced Thermal Conductivities

Xia Cai, Yiming Zhang, Zejiao Shi, Ying Chen, Yujie Xia, Anran Yu, Yuanfeng Xu, Fengxian Xie, Hezhu Shao, Heyuan Zhu, Desheng Fu, Yiqiang Zhan, and Hao Zhang**

Discovery of Lead-free Perovskites for High-performance Solar Cells via Machine Learning: Ultrabroadband Absorption, Low Radiative Combination and Enhanced Thermal Conductivities

Xia Cai,[1,2] Yiming Zhang,[1,3] Zejiao Shi,[1,2] Ying Chen,[1] Yujie Xia,[1,3] Anran Yu,[1,2] Yuanfeng Xu,[4] Fengxian Xie,[1] Hezhu Shao,[5] Heyuan Zhu,[1,3] Desheng Fu,[6] Yiqiang Zhan,[1,2,*] and Hao Zhang[1,3,7,†]

[1]*School of Information Science and Technology, Fudan University, Shanghai 200433, China*

[2]*Center of Micro-Nano System, School of Information Science and Technology, Fudan University, Shanghai 200433, China*

[3]*Key Laboratory of Micro and Nano Photonic Structures (MOE) and Department of Optical Science and Engineering, Fudan University, Shanghai 200433, China*

[4]*School of Science, Shandong Jianzhu University, Jinan 250101, Shandong, China*

[5]*College of Electrical and Electronic Engineering, Wenzhou University, Wenzhou, 325035, China*

[6]*Department of Electronics & Materials Sciences, Faculty of Engineering, & Department of Optoelectronics and Nanostructure Science, Graduate School of Science and Technology, Shizuoka University, Hamamatsu, 432-8561, Japan*

[7]*Yiwu Research Institute of Fudan University, Chengbei Road, Yiwu City, Zhejiang 322000, China*

Machine learning algorithms

In our work, seven different supervised ML algorithms are adopted, i.e.,gradient boosting regression (GBR), kernel ridge regression (KRR), random forest regression (RFR), decision tree regression (DTR), kernel neighbors regression (KNR), support vector regression (SVR), and linear regression (MLR). All of these models are trained on a subset of the whole data, called training set, and then the well trained model is used to predict other new data, called test set. The input dataset is randomly split into a training set and test set in ratios of 80% and 20%. The selections of appropriate input features and ML algorithms are two important steps for achieving good prediction performance of ML models. The selection of features is also called feature engineering, which is developed to obtain the importance of each feature and build the relationship between features and property. Through feature engineering, the key mechanism determining the physical property can be known and it is helpful for the material design. The hyper-parameters search is also needed to be applied to different ML models for better performance. DTR and RFR are both based on tree arithmetic, the primary hyper-parameters are the number of trees, the maximum depth of the tree, the minimum number of samples required to split an internal node and the minimum number of samples required to be at a leaf node. For KRR model, the primary hyper-parameters are alpha, kernel type and gamma parameter. The primary hyper-parameters of KNR are the number of neighbors, weight function used in prediction and algorithm used to compute the nearest neighbors. For SVR model, the primary hyper-parameters are penalty parameter C of the error term, kernel type and kernel coefficient. Each algorithm with best hyper-parameters is trained on the same dataset and in order to evaluate the performance of each ML model, three indexes are selected to estimate the performance of prediction results: determination coefficient (R^2), mean square error (MSE) and mean absolute error (MAE), calculated by:

$$R^2 = 1 - \frac{\sum_{i=1}^{N}(y_i^{true} - y_i^{pred})^2}{\sum_{i=1}^{N}(y_i^{true} - \bar{y}_i^{pred})^2} \tag{S1}$$

$$RMSE = \sqrt{\frac{\sum_{i=1}^{N}(y_i^{true} - y_i^{pred})^2}{N}} \tag{S2}$$

$$MAE = \frac{1}{N}\sum_{i=1}^{N}\left|y_i^{true} - y_i^{pred}\right| \tag{S3}$$

N is the number of samples, y_i^{true} is the actual value, y_i^{pred} is the predicted value and $\bar{y}_i^{pred}$ is the mean value of y^{pred}.

TABLE S1. The features about elemental and material properties for the regression models of HOIDPs.

Symbol	Property	Symbol	Property
$HOMO_A$	HOMO for A site	M_i	Atomic weight
$LUMO_A$	LUMO for A site	R_i^{cov}	Covalent radius by Cordero et al.
$HOMO_A\_LUMO_A$	Difference between HOMO and LUMO	P_i^d	Dipole polarizability
P_A	Ionic polarizability for A site	E_i^{ea}	Electron affinity
P_A^a	An-isotropic polarizability for A site	χ_i	Pauling's scale of electronegativity
N_A	The number of atoms in A site	M	The sum of atom weight in formula
M_A	The weight of A site	T_i^{mp}	Melting point
T_f	Tolerance factor defined as $\frac{R_A+R_X}{\sqrt{2}(R_{\bar{B}}+R_X)}$	H_i^{fh}	Heat of formability
O_f	Octahedral factor defined as $\frac{R_{\bar{B}}}{R_X}$	R_i^{vdw}	Van der Waals radius
R_i^{ion}	Ionic radii	R_i^{metal}	Single-bond metallic radius
E_i^{ip}	First ionization energies	E_i^{pa}	Proton affinity
R_i^{s+p}	Sum of s and p pseudo-potential orbital radii	N_i^{men}	Mendeleev's number
N_i^{atom}	Atom number	N_i^{period}	Period in the periodic table
R_i^{atom}	Atomic radius	N_i^{proton}	Number of protons
V_i	Atomic volume	T_i^{bp}	Boiling point
H_i^{eh}	Evaporation heat	D_i	Thermal conductivity
$R_{\bar{B}}^{ion}$	Average of B-site ionic radius	R_i^{ion}/R_j^{ion}	Ratio between two effective ionic radii
$R_i^{ion}+R_j^{ion}$	Sum of two effective ionic radii	$R_i^{ion}-R_j^{ion}$	Difference between two effective ionic radii

TABLE S2. Best values of ζ for the ground state of neutral atoms in X site of HOIDPs

element	$2s$	$2p$	$3s$	$3p$	$4s$	$4p$	$5s$	$5p$
F	**2.5638**	**2.5500**						
Cl	5.7152	6.4966	**2.3561**	**2.0387**				
Br	12.8217	15.5282	6.7395	6.5236	**2.6382**	**2.2570**		
I	39.067	48.847	34.787	34.841	25.297	24.030	**13.404**	**11.612**

TABLE S3. Comparison of the experimentally reported band gap values of HOIDPs with predicted HSE band gap values by our model.

Formula	experiment	HSE-prediction	Reference
$(CH_3NH_3)_2KYCl_6$	5.04	4.54	[1]
$(CH_3NH_3)_2KBiCl_6$	3.04	3.4	[2]
$(CH_3NH_3)_2AgSbI_6$	1.93	1.79	[3]
$(CH_3NH_3)_2AgBiBr_6$	2.02	2.48	[4]
$(CH_3NH_3)_2TlBiBr_6$	2.16	2.19	[5]

TABLE S4. Lattice parameters of 12 selected HOIDPs.

Formula	a	b	c	$\alpha(^\circ)$	$\beta(^\circ)$	$\gamma(^\circ)$
$(CH_3NH_3)_2AgAlBr_6$	8.28	10.37	7.29	89.82	90.00	90.00
$(CH_3NH_3)_2AgGaBr_6$	8.24	10.51	7.29	89.43	89.99	90.00
$(CH_3NH_3)_2AgInBr_6$	8.21	10.80	7.44	90.38	90.00	89.99
$(HC(NH_2)_2)_2AgGaBr_6$	8.75	12.78	6.48	91.08	91.44	90.54
$(C_2NH_6)_2AgTiBr_6$	7.93	12.10	7.36	90.87	79.59	87.34
$(C_2NH_6)_2AgAlBr_6$	7.89	12.16	7.35	90.46	79.00	87.18
$(C_2NH_6)_2AgInBr_6$	8.03	12.09	7.47	91.42	81.91	87.57
$(C_2OH_5)_2AgAlBr_6$	7.69	11.72	7.33	93.31	93.25	84.89
$(C_2NH_6)_2TiTiBr_6$	7.85	10.76	7.28	91.04	89.75	89.78
$(C_2NH_6)_2TiMnBr_6$	7.48	12.11	7.03	90.97	86.74	88.43
$(C_2NH_6)_2TiZnBr_6$	8.34	11.49	7.48	93.79	87.36	85.68
$(C_2NH_6)_2TiGeBr_6$	7.95	11.86	7.41	91.90	86.65	86.17

TABLE S5. Calculated density (ρ), bulk modulus (B), shear modulus (G), longitudinal (v_L), transverse (v_T) and average (v_m) sound velocities, Grueneisen parameter (γ), hole effective mass m_h^* and electron effective mass m_e^* in x/y/z direction and corresponding average for 12 selected HOIDPs.

Formula	ρ (g/cm^3)	B (Gpa)	G (Gpa)	v_L (m/s)	v_T (m/s)	v_m (m/s)	γ	$m^*_{hx}/m^*_{hy}/m^*_{hz}/m^*_h$	$m^*_{ex}/m^*_{ey}/m^*_{ez}/m^*_e$
$(CH_3NH_3)_2AgAlBr_6$	3.60	72.38	54.81	6358.24	3903	4307.8	1.28	6.23/2.16/1.04/1.89	0.41/0.33/0.38/0.37
$(CH_3NH_3)_2AgGaBr_6$	3.79	69.39	52.58	6065.95	3724	4110.22	1.28	1.14/1.61/2.66/1.60	3.34/0.60/0.68/0.87
$(CH_3NH_3)_2AgInBr_6$	3.86	70.46	50.47	5975.95	3617	3998.02	1.33	0.82/0.35/1.10/0.60	0.55/0.33/0.41/0.41
$(HC(NH_2)_2)_2AgGaBr_6$	3.43	77.94	52.36	6562.73	3907	4325.21	1.39	17.76/1.09/5.37/2.59	0.65/1.28/0.72/0.81
$(C_2NH_6)_2AgTiBr_6$	3.46	80.44	59.10	6780.73	4131	4562.93	1.30	2.69/2.07/3.70/2.67	1.66/2.67/2.69/2.22
$(C_2NH_6)_2AgAlBr_6$	3.37	79.03	58.40	6818.69	4160	4594.36	1.30	3.88/6.76/0.58/1.41	0.33/0.38/0.34/0.35
$(C_2NH_6)_2AgInBr_6$	3.66	76.05	55.78	6413.64	3906	4314.24	1.31	2.64/5.86/0.45/1.08	0.33/0.46/0.32/0.36
$(C_2OH_5)_2AgAlBr_6$	3.57	77.68	55.96	6532.68	3960	4376.22	1.32	7.70/4.03/5.80/5.45	0.40/0.43/0.41/0.41
$(C_2NH_6)_2TiTiBr_6$	3.58	90.06	68.04	7102.09	4357	4809.26	1.28	0.58/0.08/1.15/0.20	0.23/1.75/0.08/0.17
$(C_2NH_6)_2TiMnBr_6$	3.50	89.11	65.75	7101.82	4331	4783.57	1.30	49.34/4.78/9.27/8.89	3.30/14.08/2.15/3.57
$(C_2NH_6)_2TiZnBr_6$	3.17	76.50	56.77	6926.13	4230	4671.28	1.30	0.80/0.66/0.99/0.79	2.98/2.88/2.05/2.56
$(C_2NH_6)_2TiGeBr_6$	3.29	78.38	57.36	6863.70	4177	4614.5	1.31	2.96/23.37/2.72/4.01	1.29/0.58/2.57/1.04

TABLE S6. Comparison of different tolerance factors and their related parameters.

Formula	t	$\bar{\mu}$	$\Delta\mu$	OL	$TL1$	$TL2$	$CL1$
$(CH_3NH_3)_2AgAlBr_6$	1.010	0.541	0.168	0.582	0.738	0.700	1.125
$(CH_3NH_3)_2AgGaBr_6$	0.997	0.563	0.146	0.560	0.732	0.697	1.110
$(CH_3NH_3)_2AgInBr_6$	0.969	0.613	0.096	0.510	0.719	0.692	1.078
$(C_2NH_6)_2AgInBr_6$	0.986	0.613	0.096	0.510	0.719	0.692	1.078

Generalized tolerance factor:

$$t = \frac{r_A + r_X}{\sqrt{2}\left\{(\frac{r_B + r'_B}{2} + r_X)^2 + \frac{(r_B - r'_B)^2}{4}\right\}^{1/2}}$$

Average octahedral factor:

$$\bar{\mu} = \frac{r_B + r'_B}{2r_X}$$

Octahedral mismatch:

$$\Delta\mu = \frac{\left|r_B - r'_B\right|}{2r_X}$$

$$OL = \sqrt{2} - 1 + \Delta\mu$$

$$TL1 = (0.44\bar{\mu} + 1.37)/\sqrt{2}(\bar{\mu} + 1)$$

$$TL2 = (0.73\bar{\mu} + 1.13)/\sqrt{2}(\bar{\mu} + 1)$$

$$CL1 = 2.46/\left[2(\bar{\mu} + 1)^2 + \Delta\mu^2\right]^{1/2}$$

For the stretch limit and Octahedral limit, the inequalities are $t \leq 1$ and $\bar{\mu} \geq OL$. For tilt limit, t needs to meet the requirements of $t \geq TL1$ and $t \geq TL2$. For chemical limit, $t \leq CL1$ and $\bar{\mu} \leq 1.14$. As shown in TABLE S6, $(CH_3NH_3)_2AgGaBr_6$, $(CH_3NH_3)_2AgInBr_6$ and $(C_2NH_6)_2AgInBr_6$ meet the above criteria.

The analysis on the selection rule for optical transition based on the group-theory argument is conducted. By considering the dipole approximation, the electron transition is determined by electron initial/final states in the VB/CB bands $\varphi_v(k)/\varphi_c(k)$ (i.e. $|vk>/|ck>$), and the incident radiation polarization P is denoted as perturbation. To confirm the allowed or forbidden transition of electrons, the transition probability of excited electron is defined as $W(k) \propto P \cdot |<\varphi_v(k)|\nabla|\varphi_c(k)>|^2$, whose equivalent description in group theory can be expressed as $\Gamma_{perturbation} \bigotimes \Gamma_{initial} = \sum a_f \bigotimes \Gamma_{final}$, where $\Gamma_{initial}$ and Γ_{final} represent the irreducible representations (irreps) for the initial and final states and $\Gamma_{perturbation}$ denotes the perturbation term, a_f is the coefficient of the direct product decomposition. For the four HOIDP candidates, the little group and the corresponding irreps for the initial and final states at high-symmetric points in the Brillouin zone, and the irreps for the in-plane photon polarization vectors along x or y directions within the basal plane are summarized in TABLE S7- S10. Due to the orthogonality of irreps, we can perform left direct product to both side and write the criterion for optical transition as follows,

$$\Gamma_{final} \bigotimes \Gamma_{perturbation} \bigotimes \Gamma_{initial} = non\ null \tag{S4}$$

If the result for the direct product as shown in Eq. S4 is null, the optical transition is forbidden. As shown in TABLE S7- S10, for the four selected HOIDP candidates, the transitions of electron along some high-symmetric points are allowed, and some are forbidden, which means that, not all the optical transition between valence bands and conduction bands are forbidden due to the dipole forbidden rule. For $(CH_3NH_3)_2AgAlBr_6$ as shown in TABLE S7, VBM and CBM both locate at Γ point. The little group for high-symmetric point Γ is C_{2h} group, and the irreps for initial/final electron states are even-parity A_g, but the irreps for the polarization is odd-parity B_u. Therefore, according to Eq. S4, the result of W(k) is null which means the electron transition between VBM and CBM is forbidden. However, since the little group for band-edge electrons around along Γ point, e.g. $\Gamma - Z$ line as shown in TABLE S7, is C_s group and the corresponding irreps for initial/final electron states and polarization are all A', whose direct product is *non null*, thus the optical transition is allowed. So the optical properties are still significantly large.

For $(CH_3NH_3)_2AgGaBr_6$ as shown in TABLE S8, CBM locates at Γ point and VBM locates at high-symmetric points along $A - Z$ line, which means that, the optical transition for VB electrons at Γ is forbidden, and that for VBM electrons at $A - Z$ line is allowed. Similar analysis can be employed for $(CH_3NH_3)_2AgInBr_6$ as shown in TABLE S9. For $(C_2NH_6)_2AgInBr_6$ as shown in TABLE S10, since the structure possesses low group symmetry, the little groups are all C_1, and

the dipole transitions are allowed for valence-band electrons.

TABLE S7. The k-space location of each exciton and selection rules for $(CH_3NH_3)_2AgAlBr_6$ based on group theory. The capital letter in initial state and final state column represent for the irreps of band state. The electron-hole pair excitation are taken under consideration at high symmetry points and high symmetry line.

BZ point	Initial state	Polarization	Final state	W(k)
$\Gamma(C_{2h})$	A_g	$x, y \in B_u$	A_g	$A\_g \bigotimes B_u \bigotimes A\_g = null$
$X(C_s)$	A″	$x, y \in A'$	A″	$A'' \bigotimes A' \bigotimes A'' = non\ null$
$M(C_1)$	A	$x, y \in A$	A	$A \bigotimes A \bigotimes A = non\ null$
$\Gamma(C_{2h})$	A_g	$x, y \in B_u$	A_g	$A\_g \bigotimes B_u \bigotimes A\_g = null$
$\Gamma - Z(C_s)$	A′	$x, y \in A'$	A′	$A' \bigotimes A' \bigotimes A' = non\ null$
$Z(C_s)$	A′	$x, y \in A'$	A″	$A' \bigotimes A' \bigotimes A'' = null$
$R(C_s)$	A″	$x, y \in A'$	A′	$A'' \bigotimes A' \bigotimes A' = null$
$A(C_1)$	A	$x, y \in A$	A	$A \bigotimes A \bigotimes A = non\ null$
$Z(C_s)$	A′	$x, y \in A'$	A″	$A' \bigotimes A' \bigotimes A'' = null$
$X(C_s)$	A″	$x, y \in A'$	A″	$A'' \bigotimes A' \bigotimes A'' = non\ null$
$R(C_s)$	A″	$x, y \in A'$	A′	$A'' \bigotimes A' \bigotimes A' = null$
$M(C_1)$	A	$x, y \in A$	A	$A \bigotimes A \bigotimes A = non\ null$
$A(C_1)$	A	$x, y \in A$	A	$A \bigotimes A \bigotimes A = non\ null$

TABLE S8. The k-space location of each exciton and selection rules for $(CH_3NH_3)_2AgGaBr_6$ based on group theory.

BZ point	Initial state	Polarization	Final state	W(k)
$\Gamma(C_{2h})$	B_g	$x, y \in B_u$	A_g	$B\_g \bigotimes B_u \bigotimes A\_g = null$
$X(C_s)$	A′	$x, y \in A'$	A″	$A' \bigotimes A' \bigotimes A'' = null$
$M(C_1)$	A	$x, y \in A$	A	$A \bigotimes A \bigotimes A = non\ null$
$\Gamma(C_{2h})$	B_g	$x, y \in B_u$	A_g	$B\_g \bigotimes B_u \bigotimes A\_g = null$
$Z(C_s)$	A′	$x, y \in A'$	A″	$A' \bigotimes A' \bigotimes A'' = null$
$R(C_s)$	A′	$x, y \in A'$	A′	$A' \bigotimes A' \bigotimes A' = non\ null$
$A(C_1)$	A	$x, y \in A$	A	$A \bigotimes A \bigotimes A = non\ null$
$A - Z(C_1)$	A	$x, y \in A$	A	$A \bigotimes A \bigotimes A = non\ null$
$Z(C_s)$	A′	$x, y \in A'$	A″	$A' \bigotimes A' \bigotimes A'' = null$
$X(C_s)$	A′	$x, y \in A'$	A″	$A' \bigotimes A' \bigotimes A'' = null$
$R(C_s)$	A′	$x, y \in A'$	A′	$A' \bigotimes A' \bigotimes A' = non\ null$
$M(C_1)$	A	$x, y \in A$	A	$A \bigotimes A \bigotimes A = non\ null$
$A(C_1)$	A	$x, y \in A$	A	$A \bigotimes A \bigotimes A = non\ null$

TABLE S9. The k-space location of each exciton and selection rules for $(CH_3NH_3)_2AgInBr_6$ based on group theory.

BZ point	Initial state	Polarization	Final state	W(k)
$\Gamma(C_{2h})$	A_g	$x, y \in B_u$	A_g	$A\_g \bigotimes B_u \bigotimes A\_g = null$
$X(C_s)$	A′	$x, y \in A'$	A″	$A' \bigotimes A' \bigotimes A'' = null$
$M(C_1)$	A	$x, y \in A$	A	$A \bigotimes A \bigotimes A = non\ null$
$\Gamma(C_h)$	A_g	$x, y \in B_u$	A_g	$A\_g \bigotimes B_u \bigotimes A\_g = null$
$\Gamma - Z(C_s)$	A′	$x, y \in A'$	A′	$A' \bigotimes A' \bigotimes A' = non\ null$
$Z(C_s)$	A′/A″	$x, y \in A'$	A″	$A' \bigotimes A' \bigotimes A'' = null\ /\ A'' \bigotimes A' \bigotimes A'' = non\ null$
$R(C_s)$	A″	$x, y \in A'$	A′	$A'' \bigotimes A' \bigotimes A' = null$
$A(C_1)$	A	$x, y \in A$	A	$A \bigotimes A \bigotimes A = non\ null$
$Z(C_s)$	A′/A″	$x, y \in A'$	A″	$A' \bigotimes A' \bigotimes A'' = null\ /\ A'' \bigotimes A' \bigotimes A'' = no\ null$
$X(C_s)$	A′	$x, y \in A'$	A″	$A' \bigotimes A' \bigotimes A'' = null$
$R(C_s)$	A″	$x, y \in A'$	A′	$A'' \bigotimes A' \bigotimes A' = null$
$M(C_1)$	A	$x, y \in A$	A	$A \bigotimes A \bigotimes A = non\ null$
$A(C_1)$	A	$x, y \in A$	A	$A \bigotimes A \bigotimes A = non\ null$

TABLE S10. The k-space location of each exciton and selection rules for $(C_2NH_6)_2AgInBr_6$ based on group theory.

BZ point	Initial state	Polarization	Final state	W(k)
$\Gamma(C_1)$	A	$x, y \in A$	A	$A \bigotimes A \bigotimes A = non\ null$
$X(C_1)$	A	$x, y \in A$	A	$A \bigotimes A \bigotimes A = non\ null$
$M(C_1)$	A	$x, y \in A$	A	$A \bigotimes A \bigotimes A = non\ null$
$\Gamma(C_1)$	A	$x, y \in A$	A	$A \bigotimes A \bigotimes A = non\ null$
$Z(C_1)$	A	$x, y \in A$	A	$A \bigotimes A \bigotimes A = non\ null$
$R(C_1)$	A	$x, y \in A$	A	$A \bigotimes A \bigotimes A = non\ null$
$A(C_1)$	A	$x, y \in A$	A	$A \bigotimes A \bigotimes A = non\ null$
$Z(C_1)$	A	$x, y \in A$	A	$A \bigotimes A \bigotimes A = non\ null$
$X(C_1)$	A	$x, y \in A$	A	$A \bigotimes A \bigotimes A = non\ null$
$R(C_1)$	A	$x, y \in A$	A	$A \bigotimes A \bigotimes A = non\ null$
$M(C_1)$	A	$x, y \in A$	A	$A \bigotimes A \bigotimes A = non\ null$
$A(C_1)$	A	$x, y \in A$	A	$A \bigotimes A \bigotimes A = non\ null$

TABLE S11. Dielectric electron-phonon coupling (α, athermal). Polaron effective mass (m_p^*), Feynman-model variational parameters (v and w), Kadanoff polaron relaxation time (τ, ps), Schultz polaron radius (r_f, nm), predicted (300 K) Kadanoff mobilities (μ_K, $cm^2V^{-1}s^{-1}$) and calculated mobilities induced by acoustic deformation potential scattering (μ_{ADP}, $cm^2V^{-1}s^{-1}$).

Formula	hole								electron							
	α_F	m_p^*	v	w	τ	r_f	μ_K	μ_{ADP}	α_F	m_p^*	v	w	τ	r_f	μ_K	μ_{ADP}
$(CH_3NH_3)_2AgAlBr_6$	3.35	3.41	13.4	10.0	0.06	3.2	5	134	1.48	0.47	12.1	10.8	0.15	4.9	90	2204
$(CH_3NH_3)_2AgGaBr_6$	5.54	4.28	17.5	10.7	0.03	2.2	2	1241	4.10	1.72	16.1	11.4	0.05	2.6	8	2629
$(CH_3NH_3)_2AgInBr_6$	2.80	0.91	17.3	14.1	0.09	2.8	27	729	2.32	0.57	17.0	14.4	0.11	3.1	53	7743
$(C_2NH_6)_2AgInBr_6$	2.71	1.77	11.8	9.2	0.07	3.9	12	54	1.60	0.47	11.0	9.6	0.14	5.2	81	9436
$CH_3NH_3PbI_3$	2.67	0.22	20.1	16.8	0.10	2.5	133	14367	2.39	0.16	19.9	17.0	0.12	2.7	197	4882
$Cs_2AgBiBr_6$	2.53	0.57	14.2	11.6	0.09	3.4	42	96	2.69	0.66	14.3	11.5	0.08	3.3	34	81

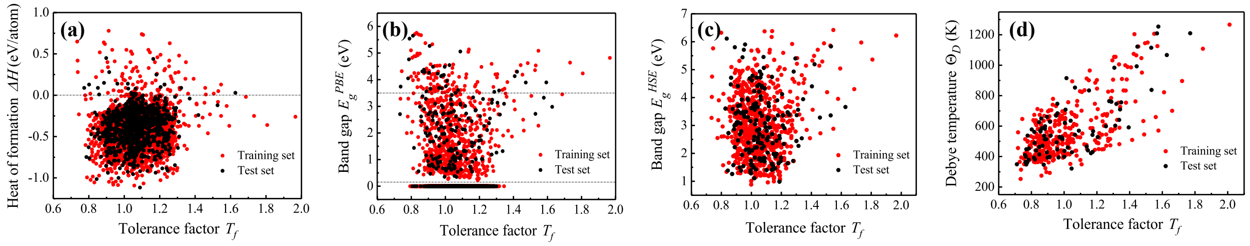

FIG. S1. Data visualization of training (red dots) and test (black dots) sets of (a) tolerance factor and heat of formation $\triangle H$, (b) tolerance factor and band gap E_g^{PBE}, (c) tolerance factor and band gap E_g^{HSE}, and (d) tolerance factor and Debye temperature Θ_D, respectively.

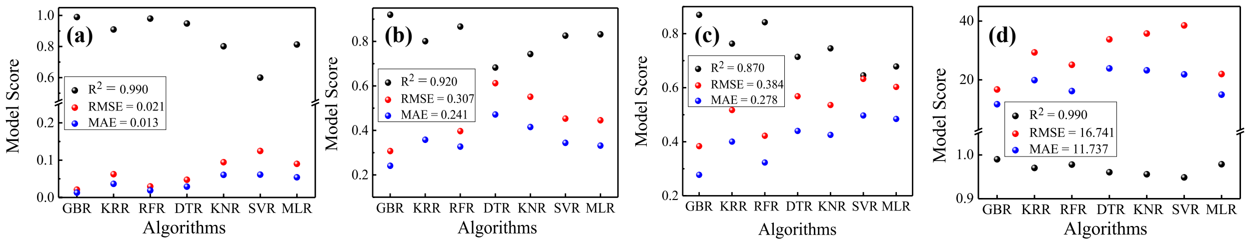

FIG. S2. The results of model selection based on seven regression algorithms (GBR, KRR, RFR, DTR, KNR, SVR and MLR) for the goals of (a) formation energy per atom ΔH, (b) band gap E_g^{PBE}, (c) band gap E_g^{HSE} and (d) Debye temperature θ_D, respectively.

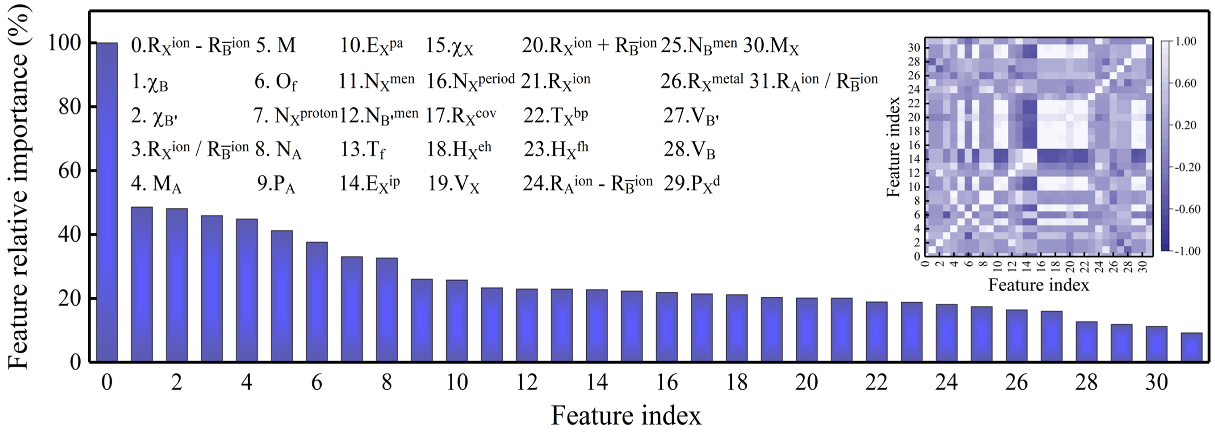

FIG. S3. Relative importance of top 32 features in PBE band-gap model. The inset is the heat map of Pearson correlation coefficient of 32 features and color bar on the right describes the values of correlation coefficient: white means positive correlation and violet means negative correlation.

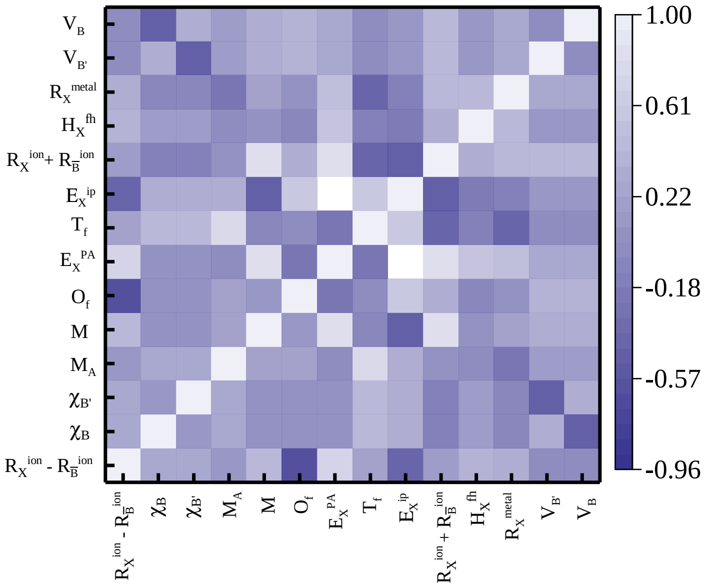

FIG. S4. The heat map of Pearson correlation coefficient of 14 chosen features after removing the redundant and uncorrelated features in PBE band-gap model.

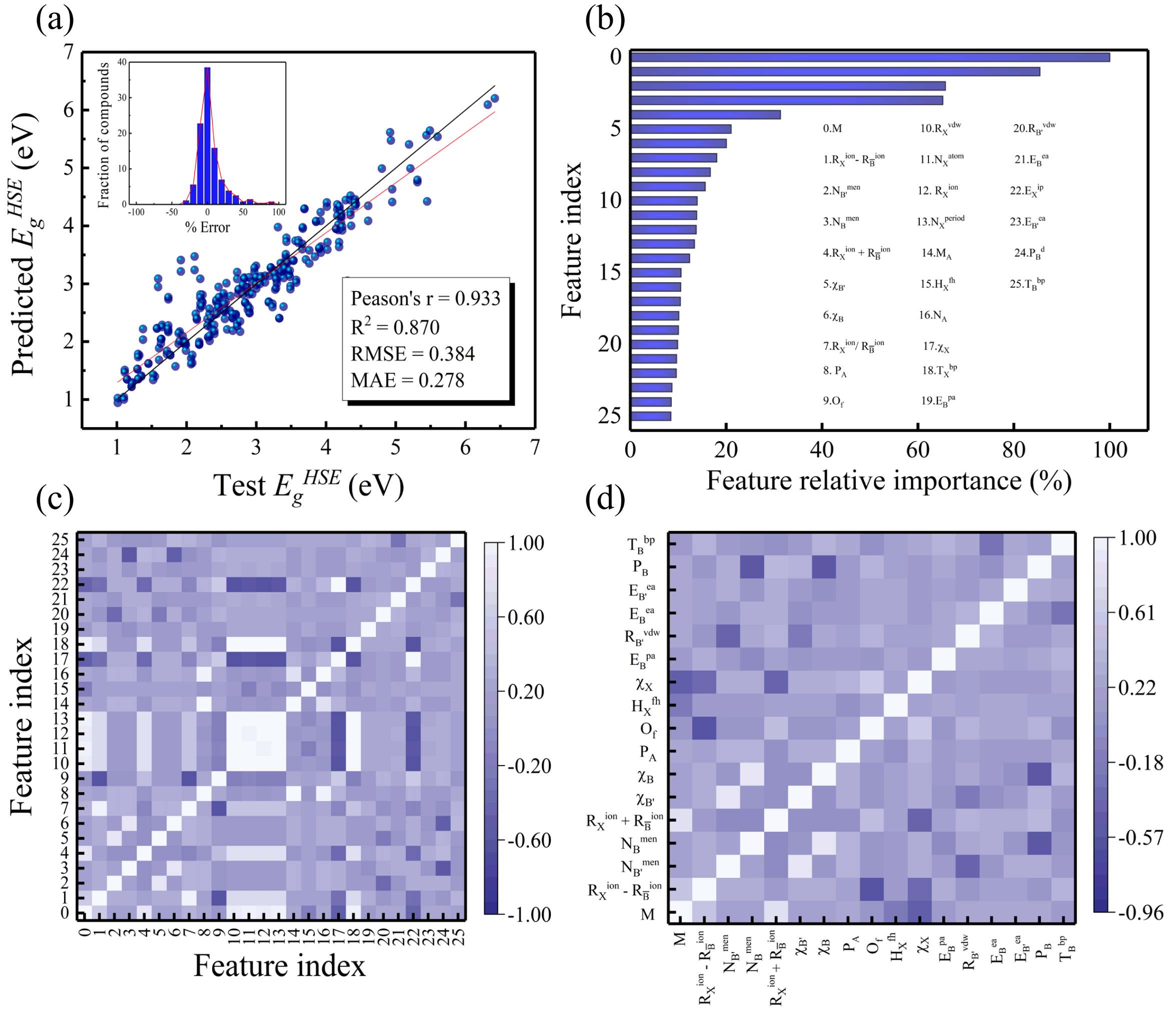

FIG. S5. The results of GBR model about band gap E_g^{HSE}. (a) The actual value of band gap E_g^{HSE} by DFT and the predicted band gap value E_g^{HSE} by ML in the test set. The ideal line is shown as red line and the fit line is shown as dark line. The inset is the fraction of compounds according to their percent error between predicted and actual values and the red curve shows the trend. (b) Relative importance of top 26 features. The heat map of Pearson correlation coefficient of (c) 26 top features and (d) 17 chosen features after removing the redundant and uncorrelated features. Color bar on the right describes the values of correlation coefficients: white means positive correlation and violet means negative correlation.

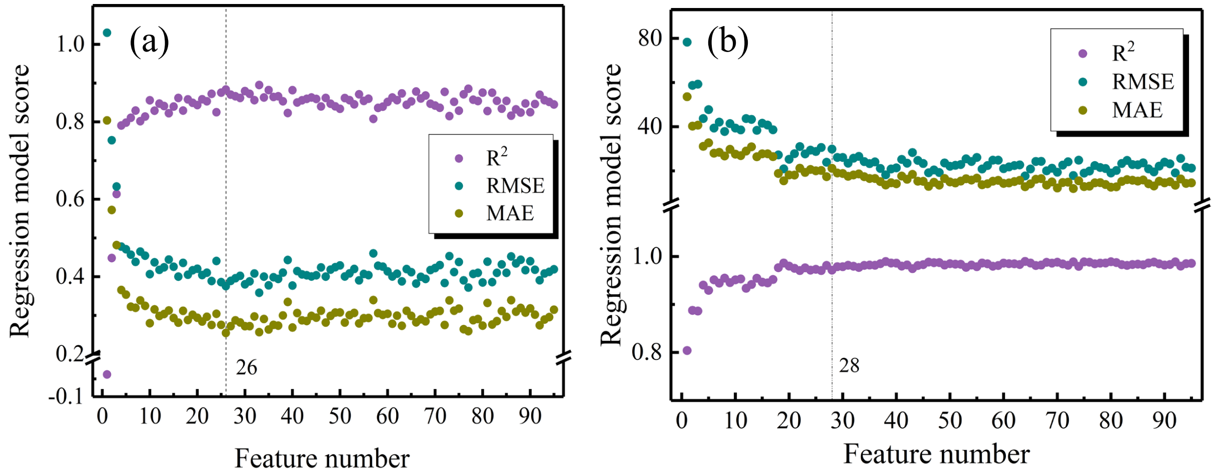

FIG. S6. Feature elimination process for (a) E_g^{HSE} model and (b) Debye temperature model, which shows the values of R^2, MSE and MAE with the number of selected features, respectively.

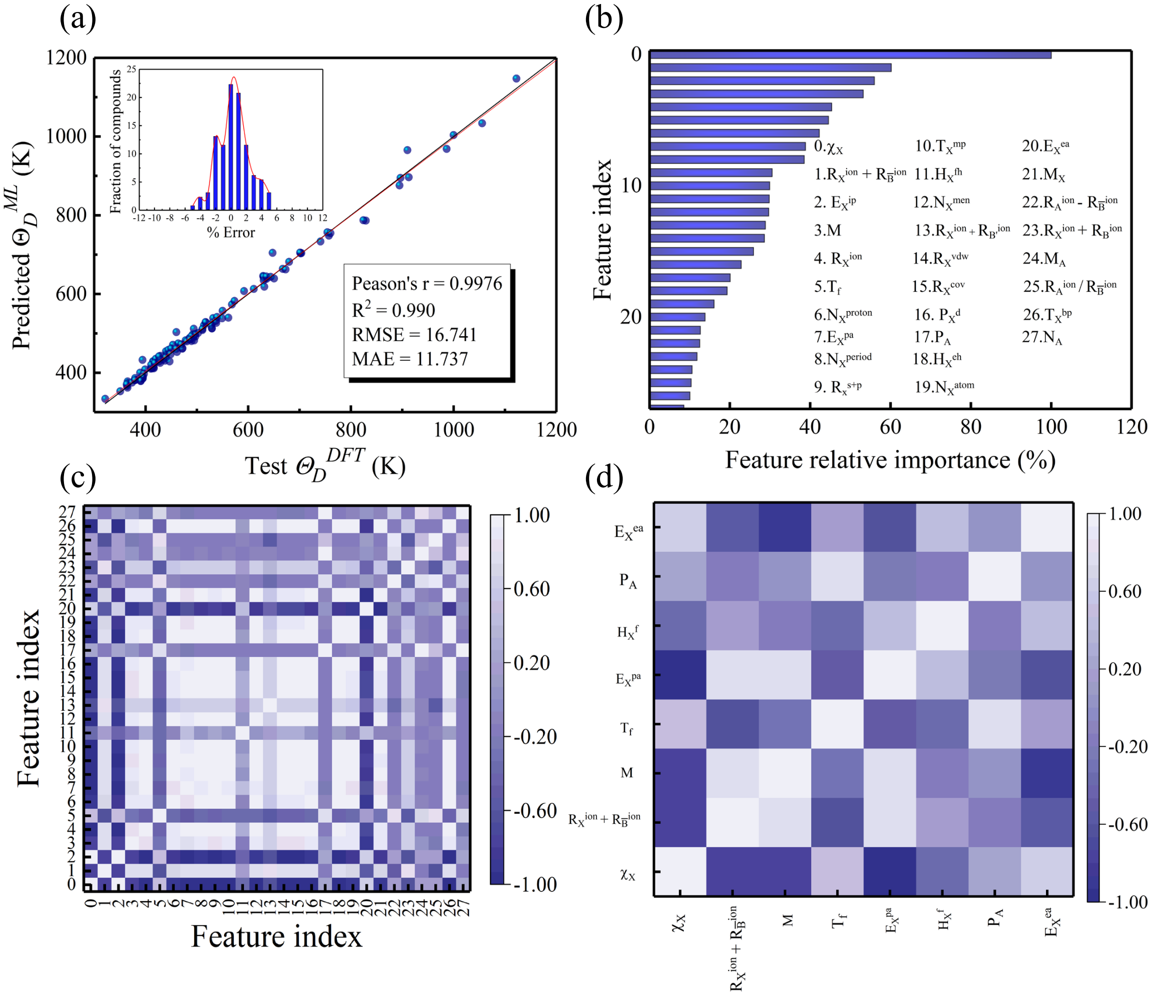

FIG. S7. (a) The fitting results of actual Debye temperature Θ_D^{DFT} and predicted Debye temperature Θ_D^{ML} obtained by gradient boosting regression. The ideal line (red line) and the fitting line (dark line) basically coincide. And Pearson coefficient (r) is 0.9976, coefficient of determination (R^2) is 0.990, mean squared error (MSE) is 16.741, and mean absolute error (MAE) is 11.737. The inset is the fraction of compounds according to their percent error between predicted Θ_D^{ML} and Θ_D^{DFT}. (b) Relative importance of top 28 features from 95 features. The heat map of Pearson correlation coefficient of (c) 28 top features and (d) 8 chosen features after feature elimination.

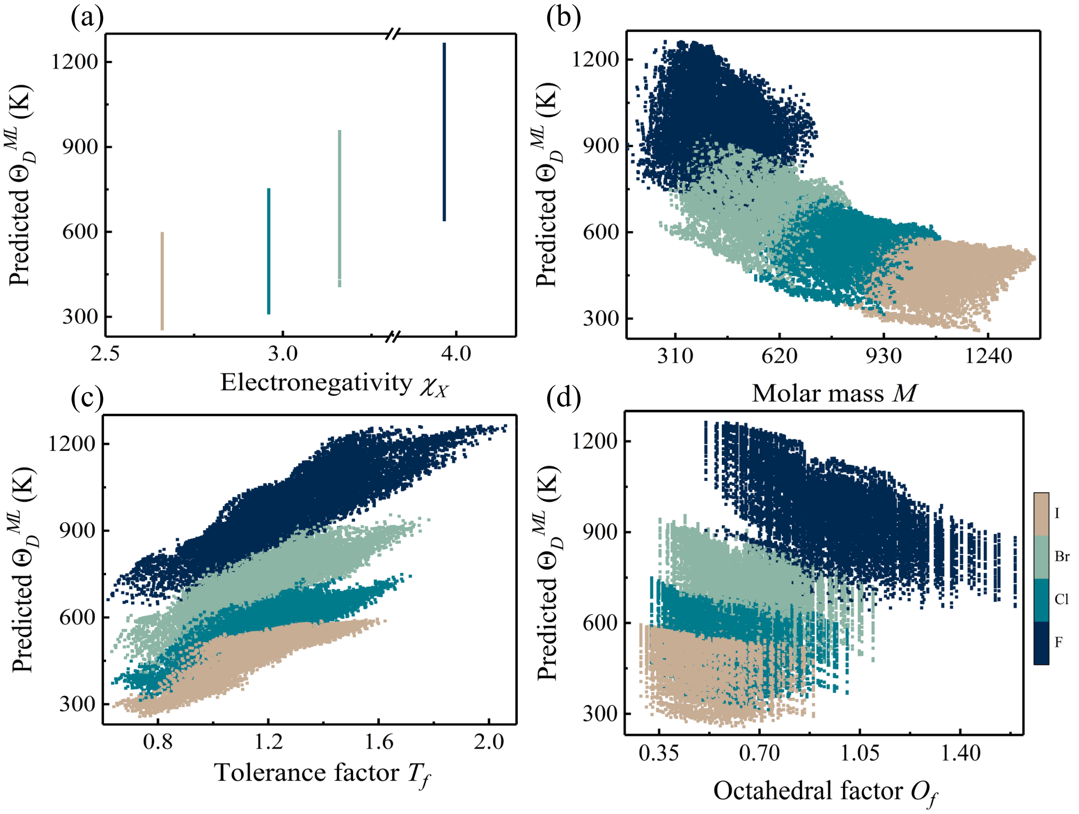

FIG. S8. Relationship visualization of prediction results with structure-property of GBR model for Debye temperature. The prediction of Debye temperature for all electrically neutral candidate HOIDPs with (a) electronegativity of X-site anion χ_X, (b) molar mass of compound M, (c) tolerance factor T_f and (d) octahedral factor O_f. Different colors represent different anions in X site of HOIDPs.

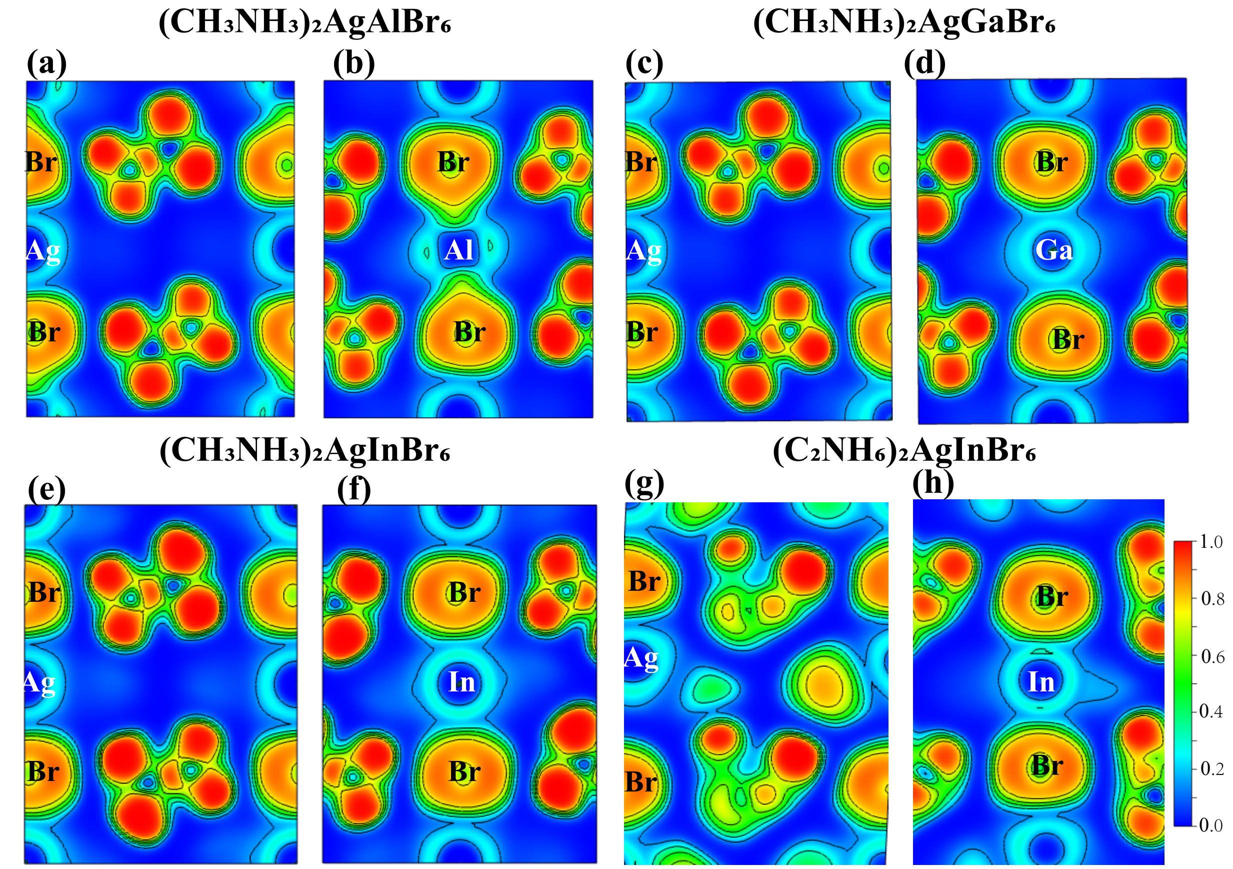

FIG. S9. Contours illustrate electron localization function (ELF), which takes a value between 0.0 and 1.0, where ELF=1.0 corresponds to the perfect localization and ELF=0.5 corresponds to the electron gas.

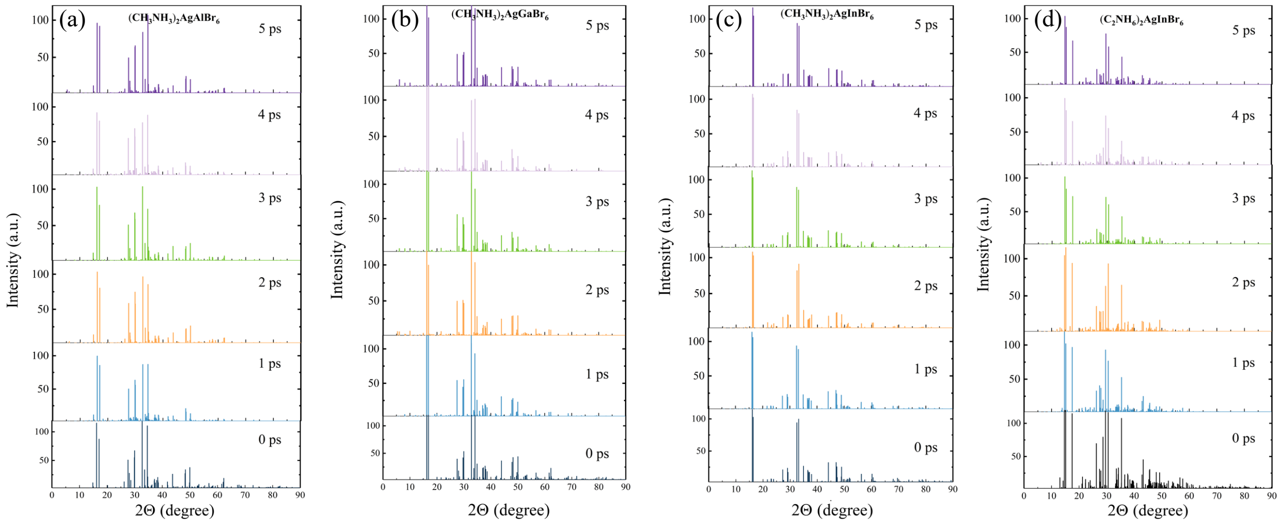

FIG. S10. Simulated XRD pattern during 5 ps AIMD simulations at room temperature for selected HOIDPs

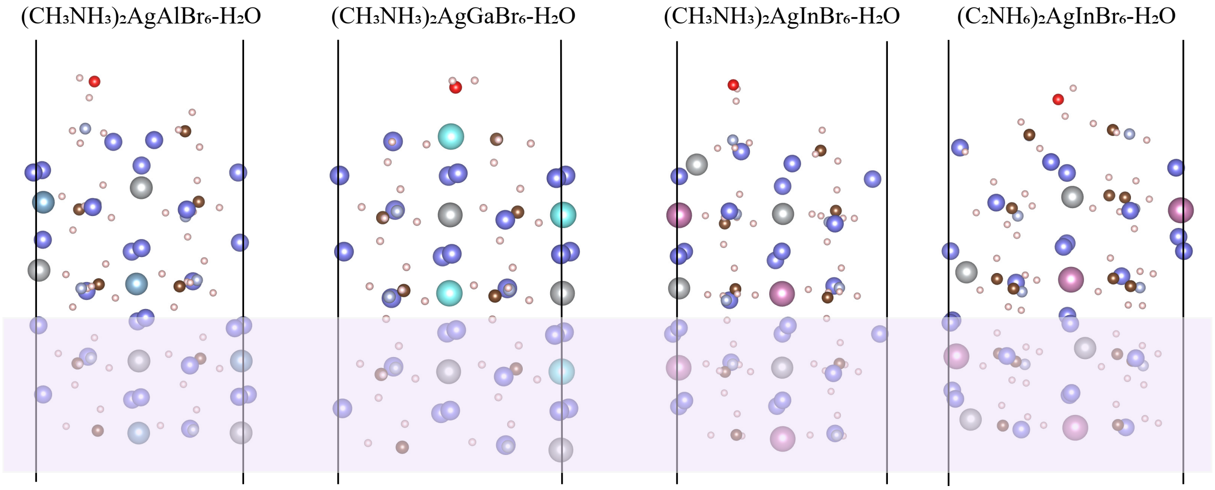

FIG. S11. Structure of four HOIDPs with one H_2O adsorbed on after optimization, where atoms are fixed in the lavender region during DFT calculation.

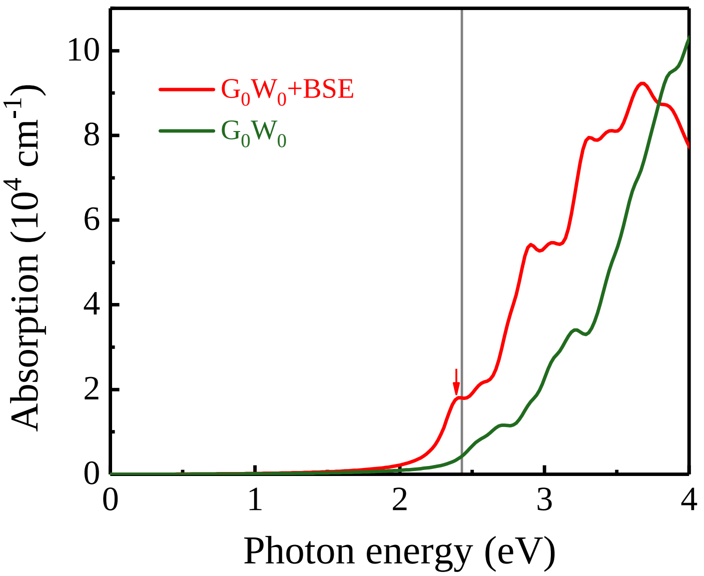

FIG. S12. Energy-dependent optical absorption calculated by G_0W_0+BSE of $CH_3NH_3PbI_3$. The structure of $CH_3NH_3PbI_3$ is from https://github.com/WMD-group/hybrid-perovskites/ with tetragonal system.

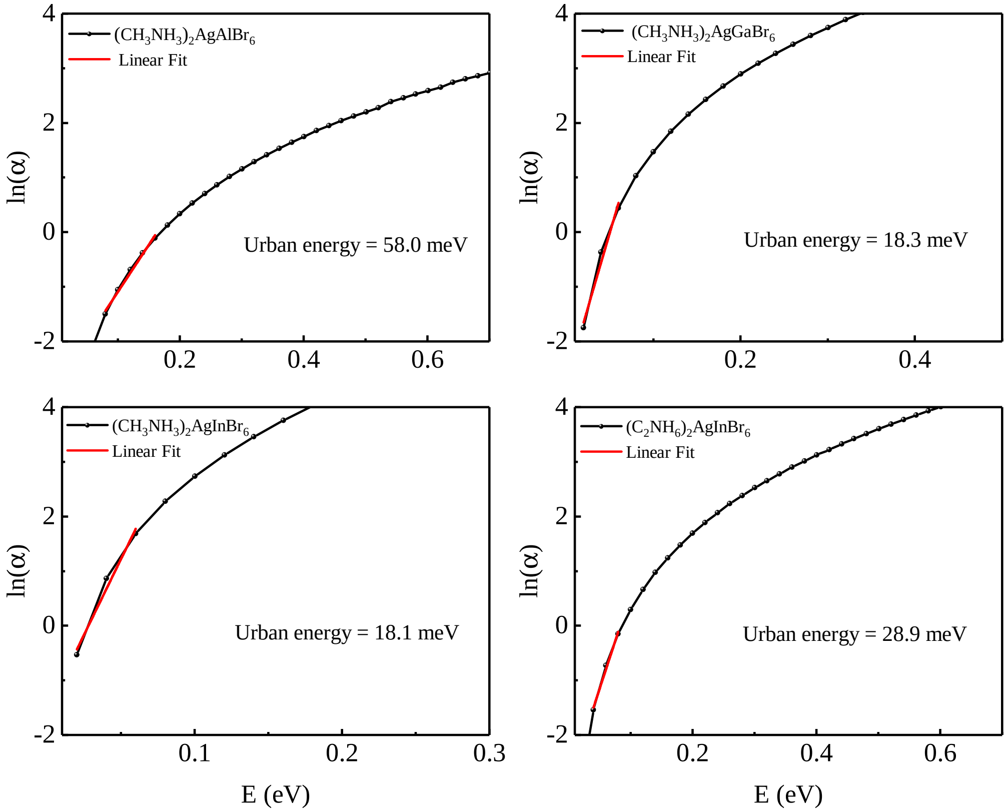

FIG. S13. Plot of Urbach energies for (a) $(CH_3NH_3)_2AgAlBr_6$, (b) $(CH_3NH_3)_2AgGaBr_6$, (c) $(CH_3NH_3)_2AgInBr_6$ and (d) $(C_2NH_6)_2AgInBr_6$.

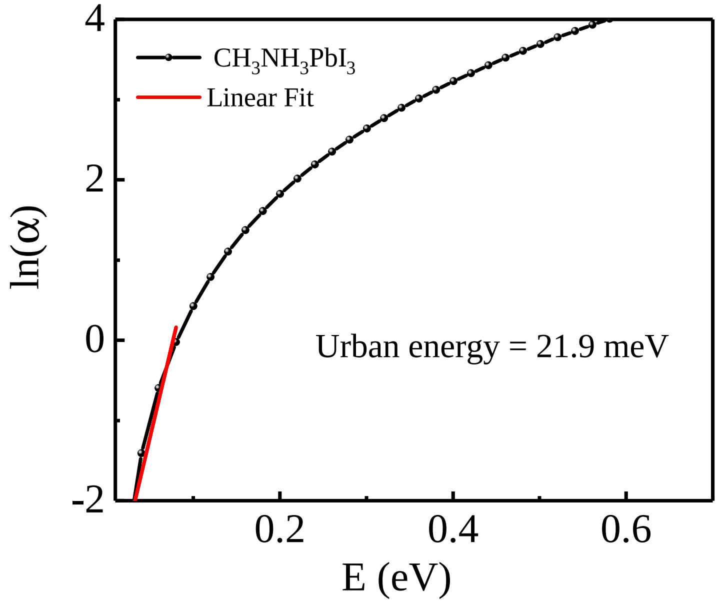

FIG. S14. Plot of Urbach energy for $CH_3NH_3PbI_3$.

[*] yqzhan@fudan.edu.cn

[†] zhangh@fudan.edu.cn

[1] Z. Deng, F. Wei, F. Brivio, Y. Wu, S. Sun, P. D. Bristowe, A. K. Cheetham, *J. Phys. Chem. Lett.* **2017**, *8*, 5015.

[2] F. Wei, Z. Deng, S. Sun, F. Xie, G. Kieslich, D. M. Evans, M. A. Carpenter, P. D. Bristowe, A. K. Cheetham, *Mater. Horiz.* **2016**, *3*, 328.

[3] Y.-J. Li, T. Wu, L. Sun, R.-X. Yang, L. Jiang, P.-F. Cheng, Q.-Q. Hao, T.-J. Wang, R.-F. Lu, W.-Q. Deng, *RSC Adv.* **2017**, *7*, 35175.

[4] F. Wei, Z. Deng, S. Sun, F. Zhang, D. M. Evans, G. Kieslich, S. Tominaka, M. A. Carpenter, J. Zhang, P. D. Bristowe, A. K. Cheetham, *Chem. Mater.* **2017**, *29*, 1089.

[5] G. García-Espejo, D. Rodríguez-Padrón, R. Luque, L. Camacho, G. de Miguel, *Nanoscale* **2019**, *11*, 16650.
